# Global Prevalence and Mental Health Outcomes of Intimate Partner Violence Among Women: A Systematic Review and Meta-Analysis

**DOI:** 10.1177/15248380231155529

**Published:** 2023-02-24

**Authors:** Sarah J. White, Jacqueline Sin, Angela Sweeney, Tatiana Salisbury, Charlotte Wahlich, Camila Margarita Montesinos Guevara, Steven Gillard, Emma Brett, Lucy Allwright, Naima Iqbal, Alicia Khan, Concetta Perot, Jacqueline Marks, Nadia Mantovani

**Affiliations:** 1 St George’s, University of London, London, UK; 2 City, University of London, London, UK; 3 King’s College London, London, UK; 4 UTE University, Ecuador, UK; 5 Anglia Ruskin University ARU, Cambridge, UK; 6 AVA (Against Violence & Abuse), London, UK; 7 Survivor Panel, London, UK

**Keywords:** domestic violence, violence exposure, battered women, mental health and violence, domestic violence and cultural contexts, cultural contexts

## Abstract

The aim of this systematic review was to assess the magnitude of the association between types of intimate partner violence (IPV) and mental health outcomes and shed light on the large variation in IPV prevalence rates between low- to middle-income countries and high-income countries. The study is a systematic review and meta-analysis. The following databases were searched for this study: Cochrane, MEDLINE, EMBASE, PsycINFO, Cumulative Index to Nursing and Allied Health Literature, and the Applied Social Sciences Index and Abstracts. The inclusion criteria for this study are as follows: quantitative studies published from 2012 to 2020 on IPV exposure in women aged 16+, using validated measures. Random effects meta-analyses and subgroup analysis exploring heterogeneity across population groups in different economic contexts are used in this study. In all, 201 studies were included with 250,599 women, primarily from high-income countries. Higher prevalence rates were reported for women’s lifetime IPV than past year IPV. Lifetime psychological violence was the most prevalent form of IPV. Women in the community reported the highest prevalence for physical, psychological, and sexual violence in the past year compared to clinical groups. Perinatal women were most likely to have experienced lifetime physical IPV. Prevalence rates differed significantly (*p* = .037 to <.001) for “any IPV” and all subtypes by income country level. Meta-analysis suggested increased odds for all mental health outcomes associated with IPV including depression (odds ratio [OR] = 2.04–3.14), posttraumatic stress disorder (PTSD) (OR = 2.15–2.66), and suicidality (OR = 2.17–5.52). Clinical and community populations were exposed to high prevalence of IPV and increased likelihood of depression, PTSD, and suicidality. Future research should seek to understand women’s perspectives on service/support responses to IPV to address their mental health needs. Work with IPV survivors should be carried out to develop bespoke services to reduce IPV in groups most at risk such as pregnant and/or help-seeking women.

## Introduction

The United Nations General Assembly made a declaration in 1993 on the “Elimination of Violence against Women” (United Nations, 1993). Nevertheless, the global burden of violence against women remains alarmingly high. Worldwide, over a quarter (27%) of women aged 15–49 years who have been in a relationship report that they have been subjected to some form of physical and/or sexual violence by their intimate partner (World Health Organization, 2021b). Intimate partner violence (IPV) refers to behavior within an intimate relationship that causes physical, sexual, or psychological harm, and includes acts of physical aggression, sexual coercion, psychological abuse, and controlling behaviors. This definition covers violence by both current and former spouses and partners (Sian Oram et al., 2022). A severe violation of human rights, IPV has human, societal, and economic costs (Rhys et al., 2019) and is increasingly recognized as a clinical and public health issue (Peterson et al., 2018; Walby & Olive, 2014). Although IPV is highly prevalent among women in the general population, as well as women attending clinical settings such as general practices, antenatal and postnatal clinics, accident and emergency services, and gynecology and family planning clinics (FRA European Union Agency for Fundamental Rights, 2014; Hawcroft et al., 2019; Makkai & Mouzos, 2004; Paulson, 2020), some evidence indicates that clinical studies report higher prevalence estimates of lifetime IPV among women than national surveys do (Beydoun et al., 2012).

A wide range of short-term and long-term physical and mental health sequelae have been associated with IPV, such as an increased risk of injury (J. L. Fanslow & Robinson, 2011; Liu et al., 2020; Thomas et al., 2021), chronic pain (Al-Modallal, 2016; Dillon et al., 2013; Loxton et al., 2017), headaches or migraine (Campbell et al., 2018; Gerber et al., 2012), and gastrointestinal and gynecological problems (Al-Modallal, 2016; Gibson et al., 2019; Karakurt et al., 2017; Stockl & Penhale, 2015). The most frequently identified IPV-related mental health consequences include posttraumatic stress disorder (PTSD) (Baker et al., 2021; Brown et al., 2020; Charak et al., 2020; Gibbs et al., 2018; Gibbs, Jewkes, et al., 2018), anxiety (Brown et al., 2020; Charak et al., 2020; Daugherty et al., 2021), depression (Ahmadabadi et al., 2020; Daugherty et al., 2021; Gibbs, Dunkle, et al., 2018; Morris et al., 2020), suicidal thoughts (Kandeger & Naziroglu, 2021; Nair et al., 2020), insomnia (Ezzati-Rastegar et al., 2020; Sanchez et al., 2016), and substance use and abuse (Alangea et al., 2018; Gibbs, Jewkes, et al., 2018; Reyes et al., 2020).

The salience of IPV as a risk factor for mental health outcomes requires further assessment. Previous systematic reviews of observational studies have identified associations between experiencing IPV and depression, suicide, PTSD, and prenatal depression (Devries et al., 2013; L. M. Howard et al., 2013; Shamblaw et al., 2019; K. Trevillion et al., 2012) with some reviews demonstrating a bidirectional relationship between IPV and mental health, and between mental health and IPV (Bacchus et al., 2018; Devries et al., 2013). A further review found increased odds of alcohol use following IPV (odds ratio [OR] = 1.25) and an increased likelihood of IPV following alcohol use (OR = 1.27) (Devries et al., 2014). None of these reviews disentangled how different types of IPV impacted on different mental health outcomes in women, nor did they explore the differential impacts of IPV on different female populations, such as perinatal, help-seeking, or community based (i.e., recruited from non-clinical settings). Therefore, this body of work does not provide a nuanced analysis that uncovers which population subgroups report higher prevalence estimates of IPV and associated mental health outcomes. Identifying groups at risk is key to allocating appropriate resources to service provision and reaching those people.

Gains made thus far in the global understanding of IPV against women come disproportionately from studies based in high-income countries. This body of knowledge is important, but may incorporate biases inherent in theory, methodologies, instrumentation, and meaning making in the global North. Although internationally there have been calls to strengthen research to learn better how to respond to violence, and calls for a global strategy and plan of action to tackle violence against women (Garcia-Moreno et al., 2015; Guedes et al., 2016), there is still limited investment in IPV research in most low- and middle-income countries (LMICs) (Temmerman, 2015), with little research examining IPV impacts on mental health. A recent study systematically reviewed the global literature on mental health outcomes and risk factors for poor mental health among Indigenous women who experienced IPV (Chmielowska & Fuhr, 2017). High rates of IPV were identified, with associated mental health morbidities among Indigenous women who experienced physical violence from their intimate partners. IPV was recognized as the strongest predictor of poor mental health. The available evidence suggests that experiences of IPV and poor mental health among Indigenous women are linked and exacerbated by poverty, discrimination, and substance use. A comprehensive evaluation of observational and experimental studies carried out in low-, middle-, and high-income countries is needed to assess the mental health impacts associated with different types of IPV in women.

To address the limitations of previous reviews, we conducted a systematic review and meta-analysis, which aimed to (1) scope the mental health outcomes examined in observational and experimental studies on IPV exposure in women; (2) quantify the prevalence of different types of IPV (physical, psychological, and sexual) in the past year and across the lifetime among different population subgroups; (3) quantify the magnitude of the association between IPV and different mental health outcomes in population subgroups; and (4) explore how the prevalence of IPV types and association of IPV with mental health outcomes found in (2) and (3) vary with respect to a country’s income level.

## Method

The research team was supported by a small Survivors’ Panel. The Survivor Panel met five times over the project life cycle to (1) shape the review’s initial and evolving focus and priorities; (2 and 3) inform the conceptualization of key issues; (4) identify key gaps in the review findings and future research priorities, and (5) contribute to dissemination and review the draft manuscript. Their involvement was supported by a third-sector organization with IPV expertise.

This systematic review and meta-analysis was registered on Prospero with the registration number (Mantovani et al., 2020) CRD42020177744. The review process followed PRISMA expanded guidelines (Page et al., 2021).

### Data Sources and Search Strategy

The search strategy comprised (a) an electronic search of six bibliographic databases and (b) forwards/backwards citation tracking. The following databases were searched: Cochrane, MEDLINE, EMBASE, PsycINFO, the Cumulative Index to Nursing and Allied Health Literature, and the Applied Social Sciences Index and Abstracts using Medical Subject Headings (MeSH) and text words from first week 2012 to 25 November 2020. The terms used to search for intimate partner violence (IPV) included domestic violence, spouse abuse gender-based violence, exposure to violence, physical abuse, rape, ((abuse* or abusive or assault* or aggress* or batter* or coerci* or control* or violen* or threat* or manipulati* or maltreat*) adj3 (physical* or sexual* or domestic or emotional* or psychological* or partner* or finan* or econom*)). The terms for mental health outcomes were adapted from a previous review (Trevillion et al., 2012) and scrutinized and modified by the Survivors’ Panel. The selection of mental health outcomes included in the review was based on the most frequent outcomes associated with IPV in the broader literature (Bacchus et al., 2018; Dillon et al., 2013). The terms included depression, anxiety, post-traumatic stress symptoms, psychological distress, suicide ideation and suicide attempt, and alcohol abuse. Authors of included articles were contacted to retrieve relevant information about their study that was either not reported or unclear from the article. An example of the search strategy is shown in Supplemental Text S1.

### Study Selection

For an illustration of the search and screening process, see Supplemental Figure S1. The study titles and abstracts were screened for relevance, and if there was insufficient abstract information to determine eligibility the full text was retrieved. Full-text articles were evaluated against the following criteria: (a) those that included non-military women who were 16 years or older and were assessed for IPV experiences (physical, psychological/emotional, and sexual) during their lifetime (lifetime IPV) or during the past year (i.e., 12 months prior to interview) using a validated IPV assessment tool; (b) those which presented the results of peer-reviewed research based on quantitative methodology that provided mental health outcome data for at least one time point. We categorized samples as clinical, perinatal, and community sample. Clinical samples could be people seeking or using care from clinical services such as primary care, drug and alcohol services, and mental health services; studies which recruited pregnant women or women in their first year postnatal were categorized as perinatal samples. Studies recruiting from non-clinical settings such as nationally or regionally representative surveys, colleges or online sources were categorized as community samples.

Language was restricted to English publications from 2012 onwards as we built on Trevillion et al.’s (2012) systematic review. Papers involving research with both men and women were included if data were disaggregated. When we identified multiple eligible papers from the same study, only the paper reporting the largest sample size was included, unless the papers were reporting on different outcomes.

Studies were excluded if they (a) included any participant aged 15 or younger or did not provide appropriate age-disaggregated data; (b) mental health outcomes were not assessed using a validated screening or diagnostic instrument or validated symptom assessments (i.e., reported clinical diagnosis without a validated instrument); and (c) reported IPV among veterans, serviceman/servicewomen, and the military. Recent systematic reviews have specifically examined IPV victimization in military populations and associated mental health outcomes (Sparrow et al., 2017, 2020; Trevillion et al., 2015).

### Data Extraction and Analysis

Downloaded titles and abstracts, and full texts were assessed by three reviewers (GdMK, CMG, and JM) against the inclusion criteria. The prime investigator (NM) independently screened 20% of each reviewer’s results at each stage (title and abstract, and full text). If the abstract did not reveal whether a paper was relevant or not, the full text was retrieved and screened. Any disagreement on eligibility between screeners was resolved by including the paper at the full-text stage. The data were extracted from final papers including the settings, sample, country, study design, independent variables, type and timing of assessments, statistical methods, and relevant findings (extracted by NM, CMG, and CW). The statistician (SW) extracted statistical data on prevalence, OR, and 95% confidence intervals (CIs). Prevalence data were extracted to respond to the second aim highlighted above, with OR used as a measure of the strength of the association between exposure to IPV and mental health. Mental health outcomes (reported as the presence or absence of different mental health disorders or symptoms) were extracted to respond to the third aim. Adjusted ORs were extracted where available; where these were not available, crude ORs were calculated from descriptive data where possible. The characteristics of included studies were summarized descriptively.

Analyses were performed using Comprehensive Meta-analysis v.3 (Biostat Inc., 2014). Meta-analyses were conducted where at least three studies provided appropriate data for the analyses described below (i.e., the minimum requirement was that three studies were available to calculate the overall pooled OR before comparing between categories). Random effects model was used throughout to calculate the pooled estimates of prevalence rates or ORs and 95% CIs for all meta-analyses to account for the substantial heterogeneity reported in similar systematic reviews (Castellvi et al., 2017; Zhang et al., 2019). Pooled estimates of prevalence and OR were reported for all groups of studies defined by timing (past year, lifetime), and type of IPV (any, physical, psychological, sexual). *I*^2^ was used to quantify statistical heterogeneity (Higgins et al., 2003), where *I*^2^ > 50% (indicating heterogeneity of considerable concern) subgroup analyses were conducted breaking down pooled estimates by population (perinatal, community, and help-seeking) and moderator analysis by World Bank income category (low, lower-middle, upper-middle, and high) to test whether these two variables explained significant clinical heterogeneity. Cochran’s Q-test (Borenstein & Higgins, 2013) was used to test for the differences between subgroups. Sensitivity analyses were conducted to test the robustness of pooled estimates. When estimating pooled prevalence, we explored the type of study design as a source of methodological heterogeneity, and whether ORs were adjusted or crude. Study designs were also tested as possible sources of methodological heterogeneity in meta-analyses of OR. If the sensitivity analyses systematically indicate that reported pooled parameter estimates vary by these methodological factors, confidence in the robustness of the results would be weakened.

### Assessment of Study Quality

Considering the wide variety of study designs, the integrated criteria for the review of multiple study designs ICROMS (Zingg et al., 2016) was used to assess study quality. This tool consists of two parts: (i) a list of quality criteria specific to different study designs (e.g., randomized controlled trials [RCTs,] cohort studies), and criteria pertinent across all study designs via a scoring system; and a “decision matrix,” which enables the assessment of the robustness of studies by identifying minimum scores consistent with study type. Studies were assessed for seven dimensions: clear aims and justification; managing bias in sampling or between groups; managing bias in outcome measurements and blinding; managing bias in follow-up; managing bias in other study aspects; analytical rigor; and managing bias in reporting/ ethical considerations. Each criterion was evaluated on a three-point scale (2 = criterion met; 1 = unclear; 0 = criterion not met). Two co-authors (NM and CW) independently assessed 25% of included articles with any disagreements resolved through discussion. These co-authors then each assessed half of the remaining 75% of articles. While no studies were excluded on the basis of quality, it is important to consider the strength of the evidence in light of the overall quality of the evidence base.

## Results

### Key Characteristics of Included Studies

**Figure 1.**
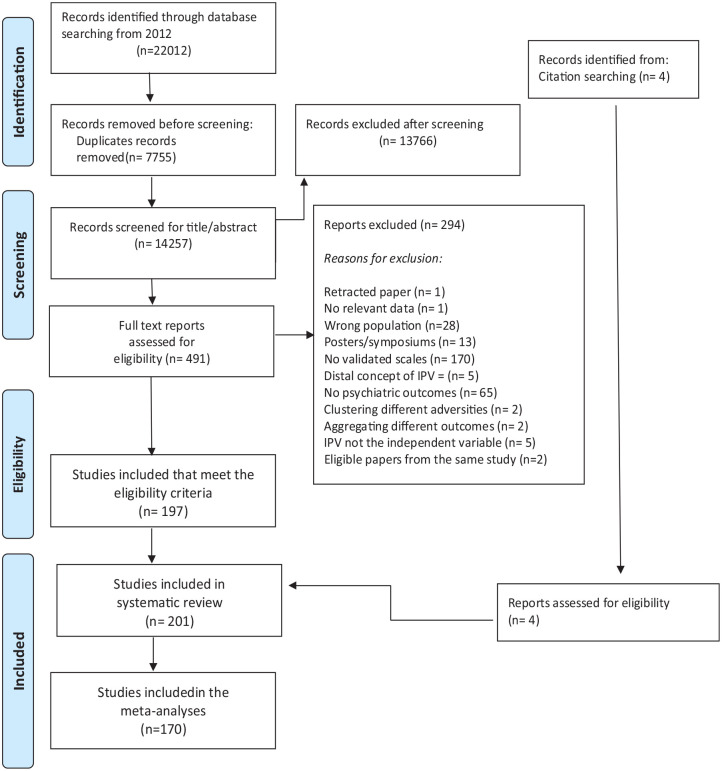
PRISMA 2020 flow diagram. Source: Matthew J Page et al. BMJ 2021; 372:bmj.n71

As illustrated in Figure 1. database searches resulted in 14,257 initial records, with 13,766 excluded following title and abstract screening. We assessed 491 full texts for eligibility, 290 were excluded. This meant that *k* = 201 studies met all eligibility criteria, with a total of 301,182 participants (men and women). Key characteristics of the included studies are summarized in Supplemental Table S1.

### Participants

Study sizes ranged from 14 to 52,509 participants, with a median of 435. Together, the studies included 250,599 women. Of 201 studies; 134 recruited participants from clinical settings/with a clinical diagnosis, 67 recruited from the community. Of those 134 studies recruiting a clinical sample, the majority (*k* = 68) were with perinatal women while the remaining recruited women exposed to IPV, or other clinical population such as help-seeking individuals (recruited from non-clinical settings). In all, 27 studies recruited a mixed adult sample from which women-only data were used.

### Design and Setting

Four different types of study design were used; 149 surveys, 42 cohort studies, seven RCTs, and three case–control studies. The studies were conducted across 46 countries. The majority were conducted in the United States (*k* = 69), with 12 in Brazil; eight in Australia and South Africa; seven each in Bangladesh, China, and India; six in Canada and Tanzania; five in Spain; four in Thailand, Hong Kong, Turkey, and the UK; three in Belgium, Korea, and Vietnam; two in Japan, Kenya, Greece, Jordan, Nepal, Nigeria, Pakistan, Peru, Portugal, and Rwanda; and one each in Sweden, Austria, Bolivia, Cameroon, Chile, Egypt, Ethiopia, Ghana, Iceland, Iran, Italy, Lebanon, Malawi, Malaysia, Mexico, Mozambique, Burma, and Syria. Three studies were multi-site across different countries and/or states: one set in Kenya and Zambia, another set in the United States, India, Nigeria, South Africa and China, and the other in Baltimore, MD, USA, St. Croix and St. Thomas, U.S. Virgin Islands. In all, 114 studies were located in high-income countries, 42 in upper-middle countries, 38 in lower-middle countries, and six in low-income countries. One of the multi-site studies was based across five countries with differing income levels: one high-income country, two lower-middle income, and two upper-middle income countries.

### Type of IPV and Measures

Studies measured different types of IPV (e.g., emotional/psychological violence, physical violence, sexual violence, controlling behavior, and harassment) with most studies measuring more than one type. Studies used 45 different measures of IPV; while most employed just one IPV measure, 40 studies utilized several. The most used measures of IPV were a version of the Conflict Tactics Scale (Straus, 1979) used by 76 studies, the World Health Organization Multi-Country Study on Women’s Health and Domestic Violence (Garcia-Moreno et al., 2006) used by 41 studies, the Abuse Assessment Screen (McFarlane et al., 1992) used by 19 studies, the Composite Abuse Scale (K. Hegarty et al., 2005) used by 17 studies, the Severity of Violence Against Women Scale (Marshall, 1992) used by 13 studies, The Psychological Maltreatment of Women Inventory (Tolman, 1999) used by 10 studies, the Sexual Experiences Survey (Koss & Gidycz, 1985) used by 7 studies, and the Index of Spouse Abuse (Hudson & McIntosh, 1981) used by 6 studies.

### Mental Health Outcome Measures

The most frequently adopted outcome measures were the Center for Epidemiologic Studies Depression Scale (Radloff, 1977) used by 49 studies, the Edinburgh Postnatal Depression Scale (EPDS; Cox et al., 1987) used by 30 studies, the Patient Health Questionnaire depression subscale (Kroenke et al., 2001) used by 18 studies, the PTSD Checklist (PCL-5) (Weathers et al., 2013) used by 19 studies, and the Beck Depression Inventory (Beck et al., 1988) used by 9 studies. The Alcohol Use Disorders Identification Test (Saunders et al., 1993) was used in 13 studies, with the Drug Abuse Screening Test (Skinner, 1982) used in four studies.

### Overall Study Quality

Our evaluation of study quality, including actual and minimum ICROMS scores for study type, was as follows: for the 42 cohort studies, ICROMS global quality scores ranged from 22 to 31 (mean = 26, ICROMS minimum score requirement = 18). For 149 cross-sectional studies, ICROMS global quality scores ranged from 15 to 26 (mean = 21, ICROMS minimum score requirement = 16). For seven RCT studies, ICROMS global quality scores ranged from 23 to 29 (mean = 27, ICROMS minimum score requirement = 22). For the three case–control studies, ICROMS global quality scores ranged from 23 to 27 (mean = 23, ICROMS minimum score requirement = 22).

## Findings

Studies measured a variety of mental health outcomes, which in the language/construction used in the original studies included the following: depression (*k* = 144), anxiety (*k* = 36), PTSD (*k* = 57), trauma (*k* = 2), psychological distress (*k* = 17), common mental health disorders (*k* = 5), alcohol abuse (*k* = 25), drug abuse (*k* = 11), suicidal ideation (*k* = 21), self-harm (*k* = 2), stress (*k* = 10), personality disorder (*k* = 2), sleep disorder (*k* = 2), mental health (*k* = 10), psychosis symptoms/experiences (*k* = 4), and others (*k* = 4) (complex PTSD, mood disturbance, symptoms of dissociation, and mental illness).

### Prevalence of “Any IPV” and Type of IPV in Women by Population Subgroup

#### Past year

The prevalence of having experienced any IPV in the past year was 24.2% (95% CI [20.4%, 28.4%], *k* = number of studies = 86, *I*^2^ = 99.5%). Pooled prevalence rates varied by population subgroups (*Q* = 6.1 *p* = .047) with women in the community reporting the highest prevalence at 31.6% (95% CI [24.0, 40.4%], *k* = 27, *I*^2^ = 99.7%), compared to women in clinical settings. Prevalence rates were 25.1% for help-seeking women (95% CI [14.9%, 39.1%], *k* = 13, *I*^2^ = 98.4%) and 20.2% for perinatal women (95% CI [15.9%, 25.2%], *k* = 46, *I*^2^ = 99.2%).

Psychological violence was the most prevalent type of IPV in the past year at 27% (95% CI [22.1%, 32.4%], *k* = 72, *I*^2^ = 99.6%), although prevalence did not vary by population (*Q* = 2.8, *p* = .249). The lowest prevalence was reported for sexual IPV at 10.1% (95% CI [7.6%, 13.2%], *k* = 65, *I*^2^ = 99.4%), with a pooled prevalence of 15.7% for physical violence (95% CI [12.8%, 19.1%], *k* = 80, *I*^2^ = 99.4%). Both physical and sexual IPV had prevalence rates which differed by population subgroups (*Q* = 8.6 *p* = .013 and *Q* = 7.2 and *p* = .027, respectively). Physical IPV was most reported by help-seeking women at 23.0% (95% CI [13.5%, 36.4%], *k* = 9, *I*^2^ = 98.1%) while the pooled prevalence rate for perinatal women was 12.3% (95% CI [8.9%, 16.7%], *k* = 48, *I*^2^ = 99.4%). Women in the community had the highest prevalence of sexual IPV at 17.2% (95% CI [10.3%, 27.2%], *k* = 21, *I*^2^ = 99.6%), with women in the perinatal period again having the lowest at 7.3% (95% CI [4.9%, 10.1%], *k* = 37, *I*^2^ = 99.2%).

#### Lifetime

The pooled prevalence of lifetime “any IPV” was 37.3% (95% CI [30.6%, 44.6%], *k* = 31, *I*^2^ = 99.3%) with no significant difference between population subgroups, *Q* = 1.9 *p* = .396. While lifetime psychological violence was reported by 32.8% of women overall (95% CI [23.1%, 44.0%], *k* = 24, *I*^2^ = 99.7%), prevalence rates differed significantly by population subgroups, *Q* = 9.8 *p* = .007. Women in the community had the highest pooled prevalence at 40.5% (95% CI [25.3%, 57.7%], *k* = 15, *I*^2^ = 99.8%), with lifetime psychological IPV considerably lower in help-seeking women at 15.1% (95% CI [7.8%, 24.9%], *k* = 5, *I*^2^ = 94.6%). The pooled prevalence for lifetime physical violence was estimated at 18.3% (95% CI [13.5%, 24.4%], *k* = 27, *I*^2^ = 99.4%) with perinatal women reporting the highest pooled prevalence at 28.8% (95% CI [19.9%, 39.7%], *k* = 6, *I*^2^ = 99.0%). The lowest pooled prevalence was reported for sexual violence, at 9.6% (95% CI [7.0%, 13.0%], *k* = 22, *I*^2^ = 98.9%).

### Association Between Past Year Experience of Type of IPV and Mental Health Outcomes

We report here the estimates of the association between type of IPV in the past year among women and mental health outcomes, quantified by OR.

#### Depression

**Figure 2.**
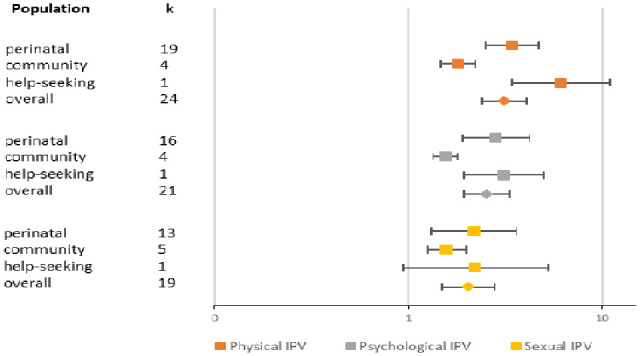
Forest plot representing pooled odds ratios of association between types of IPV in the past year and depression by population subgroup. IPV = intimate partner violence.

Estimates of the association between IPV victimization and depression were reported in studies examining physical (*k* = 24), psychological (*k* = 21), and sexual IPV (*k* = 19). We found that experiences of physical IPV were associated with the highest increased odds of depression, with a pooled OR of 3.14 (95% CI [22.42, 4.08], *I*^2^ = 85.6%), with OR = 2.54 for psychological IPV (95% CI [1.93, 3.34], *I*^2^ = 90.3%), and OR = 2.04 for sexual IPV (95% CI [1.49, 2.80], *I*^2^ = 87.1%). Population subgroups explained significant heterogeneity for both physical and psychological IPV, *Q* = 22.8 *p* < .001 and *Q* = 13.7 *p* < .001, respectively. The pooled OR for help-seeking women was highest across all violence subtypes, although this was based on one study only. Women in the community had the lowest odds of depression across violence subtypes (see Figure 2).

#### Anxiety

Estimates of the association between IPV victimization and anxiety were reported in studies examining physical (*k* = 6), psychological (*k* = 5), and sexual IPV (*k* = 4). We found that sexual violence was associated with the highest increased odds of anxiety OR = 2.34 (95% CI [1.78, 3.07], *I*^2^ = 13.0%); similarly, physical violence was associated with OR = 2.30 (95% CI [1.91, 2.77], *I*^2^ = 0%). Low heterogeneity meant that population subgroup differences were not tested. The association between psychological violence and anxiety was the lowest, with OR = 1.86 (95% CI [1.17, 2.96], *k* = 4, *I*^2^ = 61.9%).

#### Posttraumatic stress disorder

While the OR for the association between IPV types and PTSD ranged from 2.15 for sexual IPV (95% CI [0.83, 5.57], *k* = 3, *I*^2^ = 86.1%) to 2.66 for psychological IPV (95% CI [0.57, 12.36], *k* = 3, *I*^2^ = 84.1%), it should be noted that the lower limit of the 95% CI is less than 1 for all IPV types, indicating that the ORs are not significantly different from 1, *p* > .05. All these studies were with perinatal women.

#### Psychological distress

The association between types of IPV and psychological distress did not vary greatly, ranging from OR = 2.03 for psychological IPV (95% CI [1.56, 2.64], *k* = 8, *I*^2^ = 79.4%) to OR = 2.53 for sexual IPV (95% CI [2.03, 3.14], *k* = 6, *I*^2^ = 0.0%). Help-seeking women reported the highest pooled odds of psychological distress associated with physical violence and psychological violence, though there was only one study per violence subtype.

#### Suicidal ideation

Estimates of the association between physical IPV and suicidal ideation were reported in three studies. Physical IPV was associated with the highest odds of suicidal ideation, pooled OR = 4.85 (95% CI [2.93, 8.04], *I*^2^ = 31.3%). Four studies examined the association between psychological IPV and suicidal ideation, producing a pooled OR = 2.17 (95% CI [0.94, 5.02], *I*^2^ = 82.1%). However, the lower limit of the CI falls below 1.

### The Association Between Lifetime Experience of IPV and Mental Health Outcomes

We examined the association between lifetime “any IPV” and depression, psychological distress and suicidal ideation, and physical and psychological IPV type with depression.

The lifetime experience of “any IPV” was associated with a fivefold increased odd of suicidal ideation, OR = 5.52 (95% CI [1.73, 17.58], *k* = 4, *I*^2^ = 83.3%), but with high heterogeneity between studies. This heterogeneity is inflated by one study (Jina et al., 2012) which reported an OR of 79.0, considerably bigger than the other studies. This study recruited young women, aged 15–26 participating in a HIV prevention intervention. A strong association between lifetime experience of “any IPV” and suicidal ideation remained after removing this study, which reduced the pooled OR to 3.14 (95% CI [2.70, 3.66]) and substantially narrowed the CI and reduced the *I*^2^ to 0%. The lifetime experience of “any IPV” was associated with threefold increased odds of psychological distress, OR = 3.42 (95% CI [2.80, 4.18], *k* = 4), with low heterogeneity among studies, *I*^2^ = 20.6%. Estimates of the association between “any IPV” across the lifetime and depression showed an increased odds of depression, pooled OR = 2.24 (95% CI [1.70%, 2.94%], *k* = 7, *I*^2^ = 72%).

The experience of lifetime physical IPV was associated with increased odds of depression, pooled OR = 2.19 (95% CI [1.86, 2.57], *k* = 4) with low heterogeneity *I*^2^ = 8.9% between studies. The experience of lifetime psychological IPV was associated with smaller increased odds of depression, pooled OR = 1.72 (95% CI [1.05, 2.81], *k* = 5, *I*^2^ = 84.8%).

### Examination of Country Income Level as a Moderator

**Figure 3.**
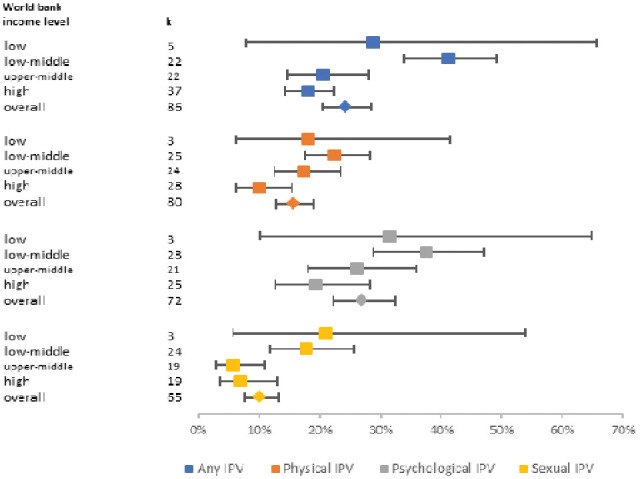
Forest plot of pooled prevalence rates of “any IPV” and IPV subtypes in the past year by World Bank Income level. IPV = intimate partner violence.

Figure 3 shows a forest plot for pooled prevalence rates of “any IPV” and type of IPV in the past year by country income levels. Prevalence rates differed significantly (*p* = .037 to < 001) for “any IPV” and all subtypes by country income levels. The highest prevalence of “any IPV” in the past year was reported in LMICs: 41.2% (95% CI [33.8, 49.1%], *k* = 22, *I*^2^ = 99.3%) compared to 18% in high-income countries (95% CI [14.3%, 22.4%], *k* = 37, *I*^2^ = 99.2%). Similar patterns were seen for lifetime IPV, highest in low or LMICs, and lowest in high-income countries. A mediation examination of perinatal studies based on country income level found the same pattern.

Country income levels explained significant heterogeneity in studies estimating the association between physical IPV in the past year and depression (*Q* = 15.8, *p* < .001): the highest pooled OR was in upper-middle income countries, OR = 6.33 (95% CI [4.67, 8.57], *k* = 3, *I*^2^ = 0.0%), and the lowest was in low-income countries, OR = 2.53. Country income levels explained significant heterogeneity in the association between psychological IPV and anxiety, *Q* = 9.0, *p* = .030, but the small number of studies within subgroups make any inferences unwise.

**Figure 4.**
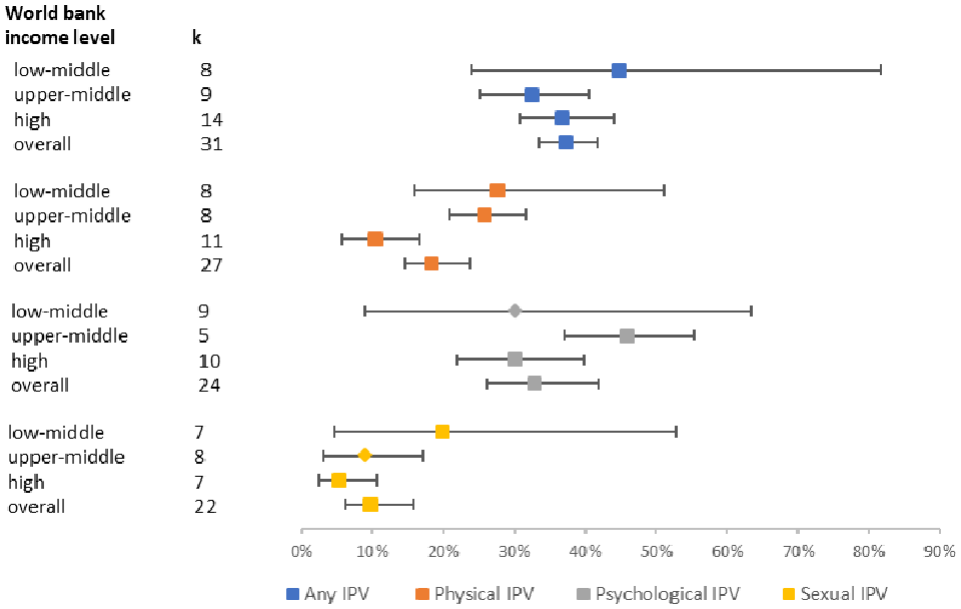
Forest plot of pooled prevalence rates of lifetime “any IPV” and IPV subtypes by World Bank Income level. IPV = intimate partner violence.

Figure 4 shows the forest plot for pooled prevalence rates of lifetime “any IPV” and type of IPV by country income levels. Lifetime prevalence of physical IPV varied across country income levels, *Q* = 7.4, *p* = .025, with highest lifetime prevalence in LMICs, 27.7%, in contrast to high-income countries, 10.3%. This pattern is repeated for sexual IPV; the pooled prevalence of lifetime sexual IPV in LMICs is 19.9%, (95% CI [15.2%, 25.5%], *k* = 7, *I*^2^ = 97.4%) and a quarter of this is in high-income countries at 5.3% (95% CI [1.7%, 15.3%], *k* = 7, *I*^2^ = 99.4%).

The association between any lifetime IPV and depression varied significantly by country income levels (*Q* = 8.9 *p* = .012), with the strongest association, OR = 3.06, in upper-middle income countries. Country income levels explained significant heterogeneity in the association between any lifetime IPV and suicidal ideation, *Q* = 17.9 *p* < .001. However, the one upper-middle income country study (Jina et al., 2012*) may be an outlier as it was a study of young women aged 16–26 from socially marginalized populations, making any inference invalid.

### Sensitivity Analysis

In all, 11 meta-analyses were conducted to estimate the pooled prevalence rates of IPV. In three of these, the study design, cross-sectional survey, or cohort was shown to significantly affect the pooled prevalence rates (*p* < .05), with surveys producing higher estimates of prevalence. In all, 24 meta-analyses were conducted to estimate the pooled OR of the association between IPV types and mental health outcome. In two of the 24, the study design, cross-sectional survey or cohort was shown to significantly affect the pooled OR (*p* < .05). Seven of the 24 studies involved in the meta-analysis were surveys. The type of OR was also investigated as a source of methodological heterogeneity. In four of the 24 meta-analyses, the adjusted ORs were significantly higher than the crude OR. Three out of 24 meta-analyses reported adjusted OR.

## Discussion

This systematic review and meta-analysis synthesized primary data from published research papers (2012–2020) on the prevalence of lifetime and past year IPV among women. It also synthesized data on the associations between IPV exposure and mental health outcomes such as depression, anxiety, PTSD, psychological distress, and suicidal ideation. The novel contributions of this work are fourfold. First, this synthesis is composed of studies using multiple research designs in low-, middle-, and high-income countries. Conclusions drawn from previous reviews have been based primarily in high-income countries, or specific global regions, limiting their generalizability. Second, this synthesis adds to the literature evidence for lifetime and past year type of IPV in both clinical and community populations. Third, this synthesis delineates the effects of both lifetime and past year type of IPV and associated mental health outcomes in population subgroups and across socioeconomic settings. Fourth, the large number of studies included provided a rich dataset, allowing rigorous moderator and subgroup analyses.

### Summary of the Main Prevalence Findings

Our review identified prevalence rates for both lifetime and past year IPV in studies recruiting women from community and clinical settings (perinatal and help-seeking). Our study confirms that, concerningly, lifetime and past year experience of “any IPV” experienced by women is highly prevalent in global terms. Overall, we found that nearly 4 in 10 (37.3%) women aged 16 and over had experienced “any IPV” in their lifetime, and one in four women (24%) had experienced “any IPV” in the past year. Our results indicate that psychological IPV in the past year and lifetime was the most prevalent form of IPV, while sexual IPV in the past year and lifetime was the least prevalent among women. In terms of population subgroups, our study found significant variations: women in the community are at a significantly high risk of experiencing any form of IPV and sexual IPV in the past year, and lifetime psychological IPV; help-seeking women are at a significantly high risk of experiencing physical IPV in the past year; and women in the perinatal period are at a significantly high risk of experiencing lifetime physical IPV.

We found a discrepancy in the global reporting of women’s lifetime experience of IPV. Our review reports a higher pooled prevalence rate for lifetime experience of “any IPV” than a multi-national study conducted in 81 countries. The World Health Organization (2013) recorded global lifetime prevalence of IPV, combining both physical and/or sexual IPV, among ever-partnered women aged 15 and over at 30.0%. Lower global estimates were also highlighted by a recent study (L. Sardinha et al., 2022) where 26% of ever-partnered women aged 15 years and older were estimated to have experienced physical or sexual IPV, or both, at least once in their lifetime. Sardinha and colleagues’ study also reported much lower global rates of past year experience of physical or sexual violence, or both, by a partner among ever partnered women aged 15–49 years. Neither of these studies examined psychological IPV. Despite the increasing recognition of the often invisible but impactful nature of psychological trauma, including coercion and/or control, most of the research has focused on physical IPV (FitzPatrick et al., 2022). It needs to be specified that the overlapping CI highlighted in the forest plots above make interpretation difficult. Moreover, Figures 3 and 4 meta-analysis on studies from LMICs have particularly wide CIs, indicating that those analysis results are less robust, probably due to the smaller number of studies/data available.

In comparison to other studies focusing on perinatal IPV, our study reported higher prevalence rates for physical IPV in the lifetime among women in the perinatal period. For example, the study by Román-Gálvez et al. (2021) reporting global prevalence rates of IPV in pregnancy, recorded a 9.2% pooled estimate for physical IPV. The prevalence rates of physical IPV in pregnancy ranged from 0.7% to 55.1% across 126 studies. Their lower estimates for this type of IPV might be explained by the fact that the authors measured physical IPV during pregnancy but reported it as lifetime (Román-Gálvez et al., 2021). It could also be because physical IPV sometimes involves medical treatment-seeking and/or police, and social services, making it more detectable, clearly identified, and categorized. Importantly, intrinsic in physical IPV is the psychological/emotional IPV that women experience (e.g., fear, anxiety), as abusive partners’ induction of fear is known as one of the primary mechanisms through which they achieve control (Jaquier & Sullivan, 2014). As such, categorizations of the different types of violence is overlapping rather than mutually exclusive. This systematic review found that the prevalence for IPV among help-seeking women was lower than women in the community, which can be understood in the light of the following factors reported in the literature: lack of inclusion of IPV as an exposure or an outcome in mental health research (Sian Oram et al., 2022); poor accuracy of the tools developed for healthcare settings for clinical and research use (Feltner et al., 2018); and the under-detection of violence experiences in clinical practice with service users being reluctant to disclose such experiences in the absence of direct questioning (Louise M Howard et al., 2010; Rose et al., 2011). In addition, our findings related to the higher risk of past year physical IPV exposure among help-seeking women align with those reported in the systematic review (S. Oram et al., 2013) on the prevalence of IPV among service users in a variety of mental health settings. Oram and colleagues found the median prevalence for female in-patients was 26%. Survey data from the UK have consistently reported that prevalence of IPV is particularly high in people in contact with secondary mental health services (Khalifeh et al., 2015). We were unable to draw conclusions on the extent to which people seeking help from mental health services are at greater risk than general population because none of the studies surveyed for our review included a direct comparison with a general population or other clinical comparison group.

Our data support the assumption that women tend to experience psychological IPV in greater frequency and regularity than other types of IPV (K. L. Hegarty et al., 2013; Henning & Klesges, 2003). In relation to sexual violence, there is some evidence from population-based studies that changes are occurring in the reported prevalence rates. For example, a recent study conducted in New Zealand (J. Fanslow et al., 2021), using data from two cross-sectional population-based surveys, found changes in the reported prevalence rates of sexual IPV between 2003 and 2019 with a significant decrease in the reported lifetime prevalence of sexual IPV, from 16.9% in 2003 to 13.1% in 2019. It is possible that societal actions such as changes in legislation and the introduction of prevention campaigns and programs have resulted in slow changes in perpetrator behavior. However, low rates of sexual violence may also be imputed to under-reporting/detection of sexual violence, aggravated by factors such as the sensitivity of the subject (Watts & Zimmerman, 2002); the taboo surrounding sexual violence in certain cultures and countries (Kalra & Bhugra, 2013); survivors’ perception and experience of disclosure in healthcare settings (Heron & Eisma, 2021); the fear that seeking help would lead to child protective services involvement; and the child being removed from the survivors’ home (Sohal et al., 2020), and changes in penalties, policies, and legislation (Dowds & Agnew, 2022).

Pooling of the prevalence data might be misleading. Variations on the prevalence of IPV reported in the studies surveyed can be ascribed not only to the differences in the levels of violence between settings, but also to differences in research methods, definitions of violence, sampling techniques, interviewer training and skills, and cultural and other differences that affect respondents’ willingness to reveal intimate experiences (Watts & Zimmerman, 2002). Nevertheless, by exploring how the prevalence rates vary, when pooled across selected population groups, it can help to determine whether the pooling of all prevalence data is in effect misleading. The discussion above seeks to explore the consistency of the pooled prevalence rates found.

Disparities can also be attributed to the availability of services—not just mental health—but police, social services, and charities. Clinical studies are also difficult to compare due to the heterogeneity of settings, age of the women, and again the definition of partner violence. There is, for example, lack of clarity among clinicians and researchers regarding how to assess psychological IPV because definitions of IPV, and operationalization of definitions of IPV, differ across and within disciplines and sectors reflecting the different priorities of agencies (Sian Oram et al., 2022). Data collection on IPV and other forms of violence need improvement, coordination, and cooperation among multiple agencies (e.g., health services, specialist services, criminal justice, welfare services) which currently is lacking. Therefore, when assessing these findings, it is important to note that violence against women is almost universally under-reported (Watts & Zimmerman, 2002). Multiple reasons can be found for this under-reporting such as women not recognizing that they are experiencing violence, not feeling able to report violence as they may be not in a private setting and the way IPV questions are framed by professionals. Many women do not formally report IPV because there are few consequences for perpetrators and women fear reprisals as they have nowhere else to go and systems have little ability to protect them (Evans & Feder, 2016; Hawkins & Laxton, 2014). Thus, the findings in our review might be better understood as representing the minimum levels of violence that occur.

### Summaries of Mental Health Harms of IPV

The most common mental health outcomes reported in this study were depression (70% of all included studies), PTSD (29%), and anxiety (17%). Our meta-analysis found consistent evidence that IPV exposure significantly impacts the mental health of women by increasing the risk of adverse outcomes such as depression, suicidal thoughts and attempts, anxiety, PTSD, and psychological distress. This chimes with previous research highlighting the association between IPV and the development of anxiety, PTSD, eating disorders, depression, and suicidal ideation (Bacchus et al., 2018; Dillon et al., 2013; Loxton et al., 2017; Sugg, 2015). When examining IPV types, the meta-analysis found significant associations between depression, psychological distress and suicidal ideation and any types of IPV, as well as the combined measure for “any” lifetime or past year IPV, suggesting that differential exposures to IPV impact mental health in unique ways. This points to the importance of disaggregating analyses of types of IPV in research.

The high risk of suicidality resulting from IPV reported in this review is compatible with existing research examining IPV and suicidality, indicating a consistent relationship between experience with IPV and risk of suicidal thoughts, attempts, and completion (Gibbs, Dunkle, et al., 2018; Grose et al., 2019; Kavak et al., 2018; McLaughlin et al., 2012; Wolford-Clevenger & Smith, 2017). The fivefold increased risk of suicidal ideation for “any IPV” in the lifetime and for physical IPV in the past year, reported in our review, are congruous with those highlighted in a systematic review of longitudinal studies by Devries et al. (2013). These authors found a positive association between physical and/or sexual IPV exposure and incident suicidal attempts with an estimated effect ranging from OR = 3.2–7.97. However, included in our review was Jina et al.’s (2012) study which reported an OR of 79.0, considerably bigger than the other studies, limiting comparability. Evidence suggests that socially marginalized populations of women exposed to IPV are at even greater risk of suicidality, especially among women living with HIV (Gielen et al., 2005). Gielen and colleagues found that HIV-positive women who reported lifetime or past year IPV exposure were more likely to report suicidal thoughts and attempts than (a) women who were HIV negative and exposed to IPV, or (b) women who were HIV positive and not exposed to IPV. Mechanisms that might explain this association between IPV and suicidality, based on the interpersonal theory of suicide, include hopelessness, isolation, unemployment, sleep disturbances, poor mental health, and physical illness (Van Orden et al., 2010), all of which are independently associated with IPV (Black, 2011).

The highest increase in odds of depression was associated with the experience of physical violence for both women in the perinatal period and help-seeking women. In most of the studies researching women during the perinatal period, participants were attending a health center for pregnancy care, a time in which women have high health needs for themselves and their unborn child. During these routine checks, evidence of violence and/or associated depression can be picked up by professionals who can put into place support and measures to protect women and the unborn child from IPV. Researchers have suggested that pregnancy could be a trigger for IPV. In the WHO multi-country study on women’s health and violence against women, the majority of women who reported physical abuse during pregnancy had also been beaten prior to getting pregnant (Potter et al., 2021). The coexistence of IPV and depression impacts the health and well-being of both women and their children in ways such as premature birth; low birthweight infants; neonatal and infant mortality (Campbell, 2002; Pastor-Moreno et al., 2020); and lack of mother and child bonding. The latter includes the impact of perpetrators forbidding women in the postnatal period to express their affection and care for their baby and making it difficult to take close care of their newborn after childbirth (Mazza et al., 2021). Compared to our study, previous systematic reviews reported a smaller risk of developing postpartum depression associated with physical violence (OR = 1.90) (Zhang et al., 2019), and a moderate effect size for physical abuse and prenatal depression (*r* = 0.271) (Shamblaw et al., 2019).

In our review, help-seeking women were at the highest risk for depression across all forms of violence experienced in the past year. The help-seeking women in our study were mostly women with severe mental health problems and addiction, HIV seropositive/seronegative female drug users, or incarcerated women receiving treatment for their mental health, which may have played a role in the higher risk of depression and/or risk of experiencing violence. Although our research is not making claims about causality, other researchers have provided evidence of a bidirectional relationship suggesting that women with severe mental health problems are more likely to experience violence, as well as being more likely to develop mental health problems as a consequence of violence (Khalifeh et al., 2015; Tsai et al., 2016). In their study, Tsai et al. (2016) found that depression severity was associated with a greater risk of subsequent IPV. Each 5-point difference in the EPDS was associated with a 0.9-point to 2.3-point difference in subsequent IPV risk (ß = .054). Longitudinal studies have demonstrated that IPV increases the likelihood of depression among women with no previous history of symptoms and there is also an association between depression and subsequent IPV (Devries et al., 2013). The reported evidence is suggestive of an association between IPV and incident depression (OR = 1.97), as well as an association in the reverse direction between depression and incident IPV (OR = 1.93). More than 10% of postnatal depression, for example, may be potentially attributable to IPV (L. M. Howard et al., 2013). While research has established an association between depression symptoms and IPV victimization and perpetration (S. Oram et al., 2014; Spencer et al., 2019; K. Trevillion et al., 2012), the temporal relationship between these two experiences remains unclear. Studies have focused on IPV victimization and have not examined the reciprocal relationship between depression and IPV perpetration. In part, this reflects the lack of availability of longitudinal studies that include measurement of different types of IPV victimization and/or perpetration to establish the relative order of victimization/perpetration versus depression symptoms (Chatterji & Heise, 2021).

In the studies reviewed, psychological distress was used to describe an overall measure of morbidity such as depression, anxiety, and somatization. In our study, the experience of sexual IPV in the past year contributed to the highest risk of psychological distress among women. Sexual IPV has received relatively little attention across research, policy, and practice (McOrmond-Plummer et al., 2014; Parkinson & Reid, 2014), with the exception of some important work in the 1980s and 1990s (Bergen, 1995; Finkelhor & Yllo, 1982; Russell, 1990). While there have been some limited attempts to unpack and understand this complex and hidden problem (Tarzia, 2021), sexual IPV remains, for the most part, heavily stigmatized and wrapped in silence. This was the case even though women survivors of sexual violence by an intimate partner can also be exposed to ongoing abuse (Mahoney, 1999), often alongside physical or psychological violence that can last for many years (Easteal & McOrmond-Plummer, 2009; World Health Organization, 2013). A qualitative study (Tarzia, 2021) highlighted the complexity of women’s emotional responses to sexual IPV which were intrinsically tied to their level of awareness of what constitutes abuse, as well as the feelings women had for the perpetrator and the broader patriarchal norms around heterosexual relations. They found that women struggled to identify and name sexual IPV when it took the form of non-physical coercion and demonstrated that women struggled no matter what type of sexual IPV they experienced.

### Accounting for Heterogeneity

Significant heterogeneity between study in this area of research was expected (Castellvi et al., 2017; Zhang et al., 2019). We tested population group and income level as possible sources of that heterogeneity. However, it is clear from the high levels of heterogeneity within group statistics that other factors are also at work. Studies from countries of different income levels were not evenly distributed, with a particular lack of studies from low-income countries. This affected our ability to explore income level as a moderator. However, some important patterns were identified. The large variation in IPV prevalence rates between LMICs and high-income countries reported in this review is consistent with the different social, economic, and political circumstances that are associated with IPV and limit women’s ability to leave abusive relationships, such as economic insecurity, gender inequitable norms, high levels of societal stigma, economic insecurity, discriminatory family law, and inadequate support services (L. M. Sardinha & Catalán, 2018). Currently, mental health provision and understanding of mental health problems/needs resulting from IPV are poor in LMICs, with long-term studies or research studying lifetime IPV in LMICs being relatively limited.

In addition, globally there is a mismatch between the high need for mental health care and persistent scarcity of financial resources, workforce, and infrastructure resources for mental health services, with research indicating that more than half of people with a mental health problem are not receiving treatment globally (Kohn et al., 2004). There are also large variations in mental health expenditure per capita in high-income and low-income countries, and significant differences in the presence of mental health workforces of psychiatrists, nurses, psychologists, and social workers across low-high income countries (World Health Organization, 2018). Contrary to the global North, in LMICs mental health is not often construed as a medicalized issue, meaning women exposed to IPV experiencing psychological distress may not necessarily go to a doctor or clinic to report these symptoms, but rather may turn to their religious/faith leader or to their network of friends and family (Rathod et al., 2015). “Stakeholder collaboration” (World Health Organization, 2018) with wider communities such as faith-based organizations was adopted in LMICs in recent years as a means of intersectoral integration aimed at more responsive mental health and social care services in community-based settings.

In addition, although globally IPV against women is embedded in a broader system of unequal power relations characterized by widespread gender-based discrimination, gender norms, and stereotypes (Garcia-Moreno et al., 2015), in LMICs women generally have less access to the resources and power that make avoiding, reporting, or escaping IPV possible (Collins, 2004; World Health Organization, 2013). Acute and chronic circumstances such as poverty, conflict, natural disasters, disease and infection, and lack of access to support services and legal protections are all factors that increase vulnerability to IPV (World Health Organization, 2005). Nevertheless, the lived experiences of women in LMICs are not uniform, but rather are context dependent and shaped by local norms and social structures.

## Strengths and Limitations of this Review

Several factors contributed to the quality of this study. A comprehensive search of the global literature, including citation tracking, was conducted. Double screening of a random sample of search results was employed. Our data extraction process was rigorous and thorough; a statistical expert was responsible for extracting, checking, and calculating (if necessary) quantitative data from the included papers, alongside three other reviewers. The large dataset allowed for accuracy in moderator and sensitivity analyses.

In addition, we reported studies using clinical (i.e., healthcare seeking), perinatal and community samples, as different types of studies are useful. While community samples give a better estimate of population prevalence, clinical and perinatal samples are essential for understanding the impact of IPV on health services and can inform adaptations to clinical care and health service provision. In our review, several studies in non-English-speaking countries conducted interviews in native languages with translated and validated screening tools (e.g., Chinese, Japanese), which may improve the generalizability of results. Moreover, the study provided insights on what patterns of exposure to IPV were more strongly associated with different mental health outcomes. A further strength is the involvement of experts by experience by way of a Survivors’ Panel who contributed to the review at multiple, key stages, grounding our approach, conceptualizations, and data interpretations in lived experiences.

Although we adopted thorough and comprehensive literature searches in this review, it is possible that the exclusion of non-English language studies may have limited the generalizability of our findings. Moreover, other limitations include our review being limited by the mental health outcomes that are reported in the literature, which tend to use medical constructs (e.g., depressive symptoms, suicidal ideation) and means that we were unable to explore the constructs of particular interest to our Survivor Panel, such as understanding “symptoms” as protective coping strategies and broader mental health outcomes relating to well-being. Hindering this review is the fact that studies from different income countries were not evenly distributed, affecting the analysis. The limitations of the reported analyses reflect the reliance on the availability and quality of existing data/literature. Although there has been an increase in the number of national population-based surveys with such data, there are gaps in the availability of data in some geographic regions. Furthermore, most estimates in this study are based on women’s self-reported experiences of IPV. Given the sensitive nature of the issue, the true prevalence of physical, psychological, and sexual IPV is likely to be much higher. Survey design and implementation, including interviewer training, play an important role in enabling disclosure and affect survey results (World Health Organization, 2001).

Only instances of female victims were included in the review and meta-analysis which may be considered a limitation. In addition, the included studies mostly originated from the global North and were heterogeneous in various respects, including study design, setting and measurement of exposure and outcome variables. Interpretation of results pertaining to mental health outcomes is complicated since the spectrum covered by different screening instruments varies from mild (not requiring intervention) to severe (requiring intervention). Moreover, heterogeneity, as measured by *I*^2^ remained high, probably indicating further methodological, clinical, and population heterogeneity within subgroups, settings, and study designs, demanding caution. The sensitivity analysis exploring the methodological factors of study design and adjustment of OR as sources of heterogeneity in the meta-analyses produced little evidence that these systematically impacted on the results. As would have been expected, survey studies tended to produce higher prevalence rates, but study design had less impact on meta-analyses estimating the association between IPV and mental health outcomes. In the few meta-analyses where OR adjustment did explain heterogeneity, the studies with adjusted ORs had higher ORs. This is contrary to what would normally be expected as adjustment for confounding factors more commonly minimizes the adjusted OR. Complexities inherent in identifying and defining IPV types ought to be considered; we extracted data pertaining to IPV types as reported by studies, so if a study reported physical IPV without measuring other IPV types, we took that data with the knowledge they could have explored other IPV types. We have treated type of IPV as distinct because that is how they are treated in the literature, but in reality they will be overlapping with blurred boundaries. In addition, careful interpretation of study findings should take into account the overall quality of the literature evidence used in our meta-analyses. It is worth noting that in general, study quality for over 20% of the studies fell short of expected study reporting (ICROMS) standards.

## Implications for Practice, Policy, and Research

Despite the limitations in available data, this study clearly establishes the persistently high global prevalence of intimate partner violence. Crucially, IPV is preventable; there has been a substantial increase in the body of knowledge on what works to prevent violence against women and girls in the last decade (Jewkes et al., 2021; World Health Organization, 2019).

The evidence on the association between exposure to IPV and different mental health outcomes has important implications for the delivery of interventions and services. Women affected by IPV are, by definition, uniquely disadvantaged and at risk of developing health problems (Stockman et al., 2015), generally have poorer health outcomes than unaffected women (Stubbs & Szoeke, 2021), and where children are part of the family systems where IPV is being perpetrated, this poses additional risk of significant harm to both the child and mother (Devaney, 2015; Osofsky, 2018). As a result, women who experience IPV need tailored, nuanced care that is trauma-informed (Covington, 2016), and services need support to identify women experiencing IPV both in healthcare settings (Sohal et al., 2020) and the community. Considering the poor resources mentioned earlier, the integration of perinatal mental health into maternal and child health (MCH) services ought to be considered, as this is a time during which many women are in regular contact with health services. Staff supporting women during this phase need to be trained to be sensitive to question issues that might be linked to IPV, and sensitively suggest resources that are available to them. Moreover, knowing the prevalence of IPV and trauma history in help-seeking women, mental health services should take women’s trauma histories, including current/active IPV, and have trauma-informed approaches in place. The expansion of support and care within MCH services and outside of health services (e.g., community-based services, traditional healers, religious organizations) can be effectively achieved by upskilling non mental health specialist providers to provide mental health promotion, prevention, and treatment interventions (World Health Organization, 2021a). Research, such as that from Taylor Salisbury et al. (2021), ought to be undertaken on how to integrate the community-based non-specialist workforce with existing systems and places where women would interact with support with services in the community (Taylor Salisbury et al., 2021). Ultimately, we need to work with women in the community to understand where women go to find places of support and connection and undertake research seeking to understand from women themselves what they would like to receive as services/supports and how they would like to receive them.

## Supplemental Material

sj-docx-1-tva-10.1177_15248380231155529 – Supplemental material for Global Prevalence and Mental Health Outcomes of Intimate Partner Violence Among Women: A Systematic Review and Meta-Analysis Click here for additional data file. Supplemental material, sj-docx-1-tva-10.1177_15248380231155529 for Global Prevalence and Mental Health Outcomes of Intimate Partner Violence Among Women: A Systematic Review and Meta-Analysis by Sarah J. White, Jacqueline Sin, Angela Sweeney, Tatiana Salisbury, Charlotte Wahlich, Camila Margarita Montesinos Guevara, Steven Gillard, Emma Brett, Lucy Allwright, Naima Iqbal, Alicia Khan, Concetta Perot, Jacqueline Marks and Nadia Mantovani in Trauma, Violence, & Abuse

sj-docx-2-tva-10.1177_15248380231155529 – Supplemental material for Global Prevalence and Mental Health Outcomes of Intimate Partner Violence Among Women: A Systematic Review and Meta-Analysis Click here for additional data file. Supplemental material, sj-docx-2-tva-10.1177_15248380231155529 for Global Prevalence and Mental Health Outcomes of Intimate Partner Violence Among Women: A Systematic Review and Meta-Analysis by Sarah J. White, Jacqueline Sin, Angela Sweeney, Tatiana Salisbury, Charlotte Wahlich, Camila Margarita Montesinos Guevara, Steven Gillard, Emma Brett, Lucy Allwright, Naima Iqbal, Alicia Khan, Concetta Perot, Jacqueline Marks and Nadia Mantovani in Trauma, Violence, & Abuse

sj-docx-3-tva-10.1177_15248380231155529 – Supplemental material for Global Prevalence and Mental Health Outcomes of Intimate Partner Violence Among Women: A Systematic Review and Meta-Analysis Click here for additional data file. Supplemental material, sj-docx-3-tva-10.1177_15248380231155529 for Global Prevalence and Mental Health Outcomes of Intimate Partner Violence Among Women: A Systematic Review and Meta-Analysis by Sarah J. White, Jacqueline Sin, Angela Sweeney, Tatiana Salisbury, Charlotte Wahlich, Camila Margarita Montesinos Guevara, Steven Gillard, Emma Brett, Lucy Allwright, Naima Iqbal, Alicia Khan, Concetta Perot, Jacqueline Marks and Nadia Mantovani in Trauma, Violence, & Abuse
