# Supplementary material for: Global Prevalence and Mental Health Outcomes of Intimate Partner Violence Among Women: A Systematic Review and Meta-Analysis
Source: Trauma Violence Abuse. 2023 Feb 24;25(1):494–511. doi: 10.1177/15248380231155529 (PMC10666489; doi:10.1177/15248380231155529)
Supplement: sj-docx-1-tva-10.1177_15248380231155529 – Supplemental material for Global Prevalence and Mental Health Outcomes of Intimate Partner Violence Among Women: A Systematic Review and Meta-Analysis [file sj-docx-1-tva-10.1177_15248380231155529.docx]

**Summary table**

| **Critical findings** |
| --- |
| ***Prevalence****:* Lifetime and past year experience of ‘any IPV’ experienced by women is highly prevalent globally. Overall, nearly four in ten (37.3%) women aged 16 and over had experienced ‘any IPV’ in their lifetime, and one in four women (24%) had experienced ‘any IPV’ in the past year. Psychological IPV in the past year and lifetime was the most prevalent form of IPV, whilst sexual IPV in the past year and lifetime was the least prevalent among women. We found significant variations in population subgroups: women in the community are at a significantly high risk of experiencing any form of IPV and sexual IPV in the past year, and lifetime psychological IPV; help-seeking women are at a significantly high risk of experiencing physical IPV in the past year; and women in the perinatal period are at a significantly high risk of experiencing lifetime physical IPV. Prevalence rates differed significantly (p=0.037 – p<0.001) for ‘any IPV’ and all subtypes by income country level. The highest prevalence of ‘any IPV’ in the past year was reported in low-middle income countries, 41.2% (95% CI 33.8%-49.1%, k=22, I2=99.3%) compared to a 18.0% in high-income countries (95% CI 14.3%-22.4%, k=37, I2=99.2%).  ***Mental health harms of IPV****:* Significant associations were found between depression, psychological distress and suicidal ideation and any types of IPV, as well as the combined measure for ‘any’ lifetime or past year IPV, suggesting that differential exposures to IPV impact mental health in unique ways. This points to the importance of disaggregating analyses of IPV subtypes in research.  The highest increase in odds of depression was associated with the experience of physical violence for both women in the perinatal period and help-seeking.  Help-seeking women were at the highest risk for depression across all forms of violence experienced in the past year. The experience of sexual IPV in the past year contributed to the highest risk of psychological distress among women. |
| **Implications of the review for practice, policy, and research** |
| The evidence on the association between exposure to IPV and different mental health outcomes has important implications for the delivery of interventions and services. Women who experience IPV need tailored, nuanced care that is trauma-informed and services need support to identify women experiencing IPV both in healthcare settings and the community. The integration of perinatal mental health into maternal and child health (MCH) services should be considered, as this is a time during which many women are in regular contact with health services. Staff supporting women during this phase need to be trained to be sensitive to question issues that might be linked to IPV, and sensitively suggest resources that are available to them.  Likewise, knowing the prevalence of IPV and trauma history in help-seeking women, mental health services should take women’s trauma histories, including current/active IPV, and have trauma-informed approaches in place. The expansion of support and care within MCH services and outside of health services (e.g. community-based services, traditional healers, faith-based organisations) can be effectively achieved by upskilling non mental health specialist providers to provide mental health promotion, prevention and treatment interventions.  There is a need to work with women in the community to understand where women go to find places of support and connection and undertake research seeking to understand from women themselves what they would like to receive as services/supports and how they would like to receive them. |
