# Supplementary material for: Global Prevalence and Mental Health Outcomes of Intimate Partner Violence Among Women: A Systematic Review and Meta-Analysis
Source: Trauma Violence Abuse. 2023 Feb 24;25(1):494–511. doi: 10.1177/15248380231155529 (PMC10666489; doi:10.1177/15248380231155529)
Supplement: sj-docx-2-tva-10.1177_15248380231155529 – Supplemental material for Global Prevalence and Mental Health Outcomes of Intimate Partner Violence Among Women: A Systematic Review and Meta-Analysis [file sj-docx-2-tva-10.1177_15248380231155529.docx]

**Table S1. Characteristics of the included studies**

|  | **First Author,**  **(Year)** | **Study design**  **(Country)** | **Population** | **Sample size**  **(% fe/male)** | **Measures of intimate partner violence (IPV)** | **Types of intimate partner**  **violence (IPV)** | **Mental health outcomes** |
| --- | --- | --- | --- | --- | --- | --- | --- |
| **Studies adopting clinical sample (1 – 138) (perinatal, IPV exposed women, help-seeking)** | | | | | | | |
| **1** | Baker et al. (2020) | Survey  (USA) | US incarcerated women | 112  100% female | The cumulative trauma experiences (CTEs) | Sexual violence, non-sexual violence | **Depression & PTSD**  Patient Health Questionnaire (PHQ-9), Posttraumatic Checklist for DSM-5 Civilian Version (PCL-5) |
| **2** | Charak et al. (2020) | Survey  (UK) | UK trauma exposed sample | 1051  68.4% female  31.6% male | Modified version of Life Events Checklist (LEC) | Physical violence, violence with a weapon, sexual violence | **Anxiety, depression & PTSD**  Generalized Anxiety Disorder–7 (GAD-7), Patient Health Questionnaire-9 (PHQ-9), PTSD-Checklist for DSM-5-Civilian Version (PCL-5) |
| **3** | Conway et al. (2020) | Cohort  (Australia) | Australian pregnant women | 615  100% female | Composite Abuse Scale (CAS) | Physical violence, emotional violence | **Maternal depressive symptoms**  Edinburgh Postnatal Depression Scale (EPDS) |
| **4** | Daugherty et al. (2020) | Survey  (Spain) | Spanish IPV women | 82  100% female | Composite abuse scale- short form (CAS-SF) | Psychological violence, physical violence, sexual violence | Depression, anxiety, PTSDPatient Health Questionnaire depression subscale (PHQ-9), Generalized Anxiety Disorder Scale (GAD-7), PTSD Checklist for DSM–5 (PCL-5) |
| **5** | Edhborg et al. (2020) | Survey  (Bangladesh) | Bangladeshi postpartum women | 656  100% female | WHO Multi-Country Study on Women Health and Domestic Violence Against Women | Physical violence, sexual violence, emotional violence | **Maternal depressive symptoms**  Edinburgh Postnatal Depression Scale (EPDS) |
| **6** | Ezzati-Rastegar et al. (2020) | Survey  (Iran) | Iranian pregnant women | 456  100% female | Conflict Tactics Scale (CTS) | Psychological violence, physical violence, sexual violence, financial violence | **Anxiety & depression**  General Health Questionnaire-28 (GHQ-28) |
| **7** | Fitzpatrick et al. (2020) | Cohort  (Australia) | Australian pregnant women | 1507  100% female | Composite Abuse Scale (CAS) | Emotional violence, physical violence | **Anxiety, depression, general mental health**  Edinburgh Postnatal Depression Scale (EPDS), Short For 36 (SF36) |
| **8** | Ford-Gilboe et al. (2020) | Cohort  (Canada) | Canadian women | 462  100% female | Composite Abuse Scale (CAS) | Physical violence, emotional violence, harassment | **Depressive symptoms and PTSD symptoms**  Center for Epidemiologic Studies Depression Scale Revised (CESD-R), PTSD checklist, Civilian Version (PCL-C) |
| **9** | Hou et al. (2020) | Cohort  (China) | Pregnant women in China | 813  100% female | The Short Form of the Revised Conflict Tactics Scale (CTS2S) | Physical violence, psychological violence, sexual violence | **Postnatal depression**  Edinburgh Postnatal Depression Scale (EPDS) |
| **10** | Jouriles et al. (2020) | Survey  (USA) | US Mothers seeking services from advocacy centres | 356  100% female | Victimization subscale from the Behavioural Risk Factor Surveillance System (BRFSS) | Physical violence, psychological violence, sexual violence | **Psychological distress**  Brief Symptom Inventory (BSI) |
| **11** | Kandeger and Naziroglu (2020) | Survey  (Turkey) | Turkish IPV women | 102  100% female | Severity of Violence  Against Women Scale (SVAWS) | Physical violence, psychological violence, sexual violence | **Suicidal ideation**  Suicidal Ideation Scale (SIS) |
| **12** | Kita et al. (2020) | Cohort  (Japan) | Japanese pregnant women | 562  100% female | Index of Spouse Abuse (ISA) | Physical violence, non-physical violence | **Antenatal and postnatal depressive symptoms**  Japanese version of the Hospital Anxiety and Depression Scale (HADS) |
| **13** | Manongi et al. (2020) | Survey  (Tanzania) | Pregnant women Tanzania | 1116  100% female | Modified Swahili version of the WHO Multi-Country Study on Women Health and Domestic Violence Against Women | Emotional violence, physical violence, sexual violence | **Perinatal depression**  Edinburgh Postnatal Depression Scale (EPDS) |
| **14** | McNaughton et al. (2020) | Cohort  (South Africa) | South African pregnant women | 1480  100% female | Violence Against Women Instrument (VAWI) | Physical violence, sexual violence | **Emotional distress**  Hopkins Symptoms Checklist (HSCL-25) |
| **15** | Moreira et al. (2020) | RCT  (Portugal) | Portuguese IPV women | 23  100% female | Conjugal Violence Exposure Scale (CVES) Research Version | IPV exposure | **Depression, PTSD, CPSD**  The Patient Health Questionnaire-9 (PHQ-9), ICD-11, International Trauma Questionnaire (ITQ) |
| **16** | Nair et al. (2020) | Survey  (India) | Indian women with severe mental illness (SMI) | 100  100% female | Indian family violence and control scale (IFVCS) | IPV exposure | **Suicide behaviours**  Suicide behaviours questionnaire revised (SBQ-R) |
| **17** | Reyes et al. (2020) | Survey  (USA) | Hispanic women | 150  100% female | Revised Conflict Tactics  Scale (CTS-2) | Physical violence, psychological violence, sexual violence | **Alcohol misuse and drug misuse**  Alcohol Use Disorder Identification Test (AUDIT), Drug Abuse Screening Test (DAST) |
| **18** | Roberts et al. (2020) | Survey  (UK) | UK men/women seeking treatment for gambling disorder | 204  9.3% female  90.7% male | Jellinek–Inventory for assessing  Partner Violence (JIPV) | IPV exposure | **Depression, anxiety & alcohol use**  The Patient Health Questionnaire-9 (PHQ-9), Generalised Anxiety Disorder –  (GAD-7), Alcohol Use Disorders Identification Test Consumption Questions (AUDIT-C) |
| **19** | Sapkota et al (2020) | Randomised controlled trial  (Nepal) | Nepalese pregnant women | 140  100% female | Abuse Assessment Screen (AAS) | Physical violence, sexual violence, emotional violence | **Anxiety & depression**  Hospital Anxiety and Depression Scale (HADS) |
| **20** | Signorelli et al. (2020) | Case-control  (Italy) | Italian women | 114  100% female | Revised Conflict Tactics  Scale (CTS-2) | Sexual coercion, injury, physical violence, psychological violence | **Depressive symptoms & PTSD symptoms**  Hamilton Depression Rating Scale (HDRS), Davidson Trauma Scale (DTS) |
| **21** | Tasa-Vinyals et al. (2020) | Survey  (Spain) | Spanish individuals  with severe mental illness (SMI) | 102  47.1% female  52.9% male | Traumatic Life Events Questionnaire (TLEQ) | Physical violence, psychological violence, sexual violence | **PTSD & psychotic symptoms**  Distressing Event Questionnaire (DEQ), Brief Psychiatric Rating Scale (BPRS) |
| **22** | Thananowan et al. (2020) | Survey  (Thailand) | Thai women | 400  100% female | Abuse Assessment Screen (AAS) | Physical violence, sexual violence, emotional violence | **Depressive symptoms & stress**  The Center for Epidemiologic Studies Depression Scale (CES-D), Thai version of Perceived Stress Scale (T-PSS–10) |
| **23** | Tran et al. (2020) | Survey  (Bangladesh) | Bangladesh pregnant women | 2000  100% female | WHO Multi-Country Study on Women’s Health and Domestic Violence | Controlling behaviour, sexual violence, emotional violence, physical violence | **Maternal common mental disorders**  Self-reporting Questionnaire (SRQ-20) |
| **24** | Tutty et al. (2020) | Cohort  (Canada) | Canadian women | 587  100% female | Composite Abuse Scale (CAS) | Physical violence, emotional violence, harassment | **Depression, psychological distress & PTSD**  The Center for Epidemiological Studies–Depression (CES-D-10), Symptom Checklist–10 (SCL-10), The PTSD Checklist |
| **25** | Wadji et al. (2020) | Case-control  (Cameroon) | Dyads Cameroon | 64  100% female | Revised Conflict Tactics  Scale (CTS-2) | Psychological violence, physical violence, Sexual violence, | **Anxiety & depression**  Hospital Anxiety and Depression Scale (HADS), Symptom Checklist (SCL-27-plus) |
| **26** | Watson-Singleton et al. (2020) | Survey  (USA) | African American women | 171  100% female | Index of Spouse Abuse (ISA) | Physical violence | **Alcohol abuse**  Brief Michigan Alcoholism Screening Test (bMAST) |
| **27** | Williams et al. (2020) | Survey  (USA) | US mixed online sample | 230  61.7% female  34.4% male  3.9% transgender | Revised Conflict Tactics Scale—Victimization (CTS2). The Sexual Experiences Survey, Short Form Victimization (SES-SFV) | Psychological violence, physical violence, sexual violence, Adverse Childhood Experiences | **Depressive symptoms, PTSD symptoms & opioid misuse**  The PROMIS1 Depression 8a  The PTSD Checklist for DSM-5 (PCL-5)  PROMIS Prescription Pain Medication Misuse 7a Scale |
| **28** | Chaves et al. (2019) | Cohort  (Australia) | Australian pregnant women | 52,509  100% female | Abuse Assessment Screen (AAS) | Physical violence, frightened of partner | **Postnatal depressive symptoms**  Edinburgh Postnatal Depression Scale (EPDS) |
| **29** | Islam et al. (2019) | Survey  (Bangladesh) | Bangladeshi married mothers | 426  100% female | WHO Multi-Country Study on Women Health and Domestic Violence Against Women | Psychological violence, sexual violence, physical violence | **Postpartum depression & postpartum suicidal ideation**  Edinburgh Postnatal Depression Scale (EPDS) |
| **30** | Kokka et al (2019) | Randomized controlled trial  (Greece) | Greek IPV women | 60  100% female | Women Abuse Screening Tool (WAST) | Physical violence and emotional violence | **Depressive symptom, anxiety & stress symptoms**  Beck Depression Inventory (BDI), Perceived Stress Scale 14 (PSS-14), Depression, Anxiety, and Stress Scale (DASS-21) |
| **31** | Mathew et al. (2019) | Survey  (India) | Indian women with depression | 100  100% female | Index of Spouse Abuse (ISA) | Physical violence, non-physical violence | **Depression**  Hamilton Depression Rating Scale (HDRS) |
| **32** | Riedl et al. (2019 | Survey  (Austria) | Austrian patients | 2031  53.2% female  46.8% male | Hurt-Insult-Threaten-Scream-Scale (HITS) | Physical violence, psychological violence | **Trauma-related symptoms & psychological distress**  Essen Trauma Inventory (ETI), Brief Symptom Inventory (BSI) |
| **33** | Sullivan et al. (2019) | Survey  (USA) | US IPV women | 298  100% female | Revised Conflict Tactics Scale (CTS-2), Sexual Experiences Survey (SES) Psychological Maltreatment of Women—Short Version (PMWI-S) | Physical violence, sexual violence, psychological violence | **Depression & PTSD**  Center for Epidemiologic Studies Depression Scale (CES-D), Post-traumatic Diagnostic Scale (PDS) |
| **34** | Tho Nhi et al. (2019) | Cohort  (Vietnam) | Vietnamese pregnant women | 1274  100% female | WHO Multi-Country Study on Women Health and Domestic Violence Against Women | Emotional violence, physical violence, sexual violence | **Depression**  Edinburgh Postnatal Depression Scale (EPDS) |
| **35** | Thomas et al. (2019) | Cohort  (USA) | US pregnant adolescents | 930  100% female | Revised Conflict Tactics Scale (CTS2) | IPV exposure  IPV perpetration  Bilateral violence | **Depression & anxiety**  Center for Epidemiologic Studies Depression Scale (CES-D), Generalized Anxiety Disorder Scale (GAD-7), Revised Prenatal Distress Questionnaire (PDQ) |
| **36** | Ahmad et al. (2018) | Survey  (Malaysia) | Malaysian postpartum women | 5727  100% female | WHO Multi-Country Study on Women Health and Domestic Violence Against Women | Emotional/psychological, violence, physical violence, sexual violence | **Postnatal depression**  Malay version of the Edinburgh Postnatal Depression Scale (EPDS) |
| **37** | Barcelona de Mendoza et al. (2018) | Survey  (USA) | US pregnant women | 398  100% female | Pregnancy Risk Assessment Monitoring System (PRAMS), Center for Disease Control and Prevention | Physical violence, emotional violence | **Depression, PTSD &** **pregnancy-specific anxiety**  The Edinburgh Postnatal Depression Scale (EDS) Post-traumatic checklist (PCL-5), Revised Prenatal Distress Questionnaire |
| **38** | Behnken et al. (2018) | Survey  (USA) | US non-pregnant women accessing family planning clinics | 763  100% female | Revised Conflict Tactics Scale (CTS2) | Physical violence, sexual coercion, psychological violence | **Depression, anxiety, PTSD, alcohol & drug abuse**  World Mental Health Composite International Diagnostic Interview (WMH-CIDI) |
| **39** | Bondade et al. (2018) | Survey  (India) | Indian infertile women | 100  100% female | WHO Multi-Country Study on Women Health and Domestic Violence Against Women | Psychological violence, physical violence, sexual violence | **Depression, anxiety & suicidal ideas**  Hamilton Depression Rating Scale (HAM-D; HDRS), Hamilton Anxiety Rating Scale (HAM-A) |
| **40** | Choi et al. (2018) | Cohort  (Hong Kong) | Chinese abused women | 100  100% female | Chinese version of the Abuse Assessment Screen (C-AAS) | Psychological violence, physical violence, sexual coercion | **Depressive symptoms & PTSD**  Beck Depression Inventory, version II (BDI-II), Chinese version of the Impact of Event Scale-Revised (IES-R) |
| **41** | Field et al. (2018) | Survey  (South Africa) | South African pregnant women | 376  100% female | Revised Conflict Tactics Scale (CTS2) | Emotional violence, verbal violence, physical violence, sexual violence | **Major depressive episode, anxiety disorder, alcohol and drug use & suicidal ideation**  Mini-International Neuropsychiatric Interview (MINI Plus Version 5.0.0) |
| **42** | Honda et al. (2018) | Survey  (Japan) | Japanese IPV women | 145  100% female | Japanese version of the Domestic Violence Screening Instrument  (DVSI) | Physical violence, sexual violence, psychological violence | **Severe depression and suicidality, anxiety and mood disturbance, and response to trauma**  General Health Questionnaire 30 (GHQ 30), Impact of Event Scale-Revised (IES-R) |
| **43** | Mahenge et al. (2018) | Survey  (Tanzania) | Tanzanian postpartum women | 500  100% female | WHO Multi-Country Study on Women Health and Domestic Violence Against Women | Physical violence, sexual violence | **Postpartum depression**  Patient Health Questionnaire depression subscale (PHQ-9) |
| **44** | Mandal et al. (2018) | Survey  (Australia) | Australian postnatal women | 2621  100% female | Composite Abuse  Scale (CAS) | IPV exposure | **Postnatal depression & anxiety**  Depression, Anxiety and Stress Scale (DASS-21; Lovibond and Lovibond, 1995) |
| **45** | Mittal et al. (2018) | Survey  (USA) | US women | 175  100% female | Abuse Behavior Inventory (ABI), Women’s Experiences of Battering (WEB) | Physical violence, psychological violence, sexual violence | **Depression**  Center for Epidemiologic Studies Depression Scale (CES-D) |
| **46** | Rurangirwa et al. (2018) | Survey  (Rwanda) | Rwanda pregnant women | 921  100% female | WHO Multi-Country Study on Women Health and Domestic Violence Against Women | Physical violence, psychological violence,  sexual violence | **Major depressive disorder, generalised anxiety disorder, PTSD, & suicidal ideation**  The Mini International Neuropsychiatric Interview version 5.0.0 (MINI; Sheehan, 1998) |
| **47** | Santos and Monterio (2018) | Survey  (Brazil) | Brazilian women | 369  100% female | Revised Conflict Tactics Scale (CTS2) | Psychological violence, physical violence, sexual coercion | **Symptoms of depressive anxious mood**  Self-Reporting Questionnaire (SRQ-20) |
| **48** | Tabb et al. (2018) | Survey  (Brazil) | Brazilian pregnant women | 701  100% female | WHO Multi-Country Study on Women Health and Domestic Violence Against Women | Psychological violence, physical violence, emotional violence | **Suicidal ideation**  Clinical Interview Schedule (CIS-R) |
| **49** | Tho Tran et al. (2018) | Cohort  (Vietnam) | Vietnamese pregnant women | 1274  100% female | Modified version of the WHO Multi-Country Study on Women’s Health and Life Experiences Questionnaire | Emotional violence, physical violence, sexual violence | **Postnatal depressive symptoms**  Edinburgh Postnatal Depression Scale (EPDS) |
| **50** | Woldetensay et al. (2018) | Cohort  (Ethiopia) | Ethiopian pregnant women | 4680  100% female | HITS (Hurt, Insult, Threaten and Scream) | IPV exposure | **Prenatal depressive symptoms**  Patient Health Questionnaire depression subscale (PHQ-9) |
| **51** | Yu et al. (2018) | Survey  (China) | Chinese pregnant women | 797  100% female | Abuse Assessment Screen (AAS) | Psychological violence, physical violence, emotional violence | **Prenatal depression**  Center for Epidemiologic Studies Depression Scale (CES-D) |
| **52** | Achchappa et al. (2017) | Survey  (India) | Indian HIV+ women | 99  100% female | Abuse Assessment Screen (AAS) | Psychological violence, physical violence, sexual violence | **Depression**  Beck Depression Inventory (BDI) |
| **53** | Dami et al. (2017) | Survey  (Greece) | Greek primary care sample | 142  87.3% female 12.7% male | HITS (Hurt, Insult, Threaten and Scream) | Reported as the scale; Hits, hurt, insult (threaten), scream | **Depression**  Patient Health Questionnaire depression subscale (PHQ-9) |
| **54** | Dworkin et al. (2017) | Survey  (USA) | US substance abuse patients | 219  49% female 51% male | The National Women’s Study PTSD Module (NWS-PTSD) | Sexual violence, physical violence | **PTSD**  Clinician Administered PTSD Scale (CAPs) |
| **55** | Gibbs et al. (2017) | Survey  (South Africa) | South African pregnant women | 275  100% female | World Health Organization’s (WHO) violence against women survey and modified for South Africa | Sexual violence, physical violence | **Depressive symptoms**  Patient Health Questionnaire depression subscale (PHQ-9) |
| **56** | Islam et al. (2017) | Survey  (Bangladesh) | Bangladeshi postpartum mothers | 426  100% female | WHO Multi-Country Study on Women Health and Domestic Violence Against Women | Physical violence, sexual violence, psychological violence | **Postpartum depression**  Bangla version of the Edinburgh Postpartum Depression Scale (EPDS) |
| **57** | Miller-Graff and Cheng (2017) | Survey  (USA) | US pregnant women | 101  100% female | Revised Conflict Tactics Scale (CTS2) | Physical violence, sexual violence, psychological violence | **Depressed mood, PTSD & sleep**  Center for Epidemiologic Studies Depression Scale (CES-D), PTSD Checklist for DSM–5 (PCL-5), Pittsburgh Sleep Quality Index (PSQI) |
| **58** | Oliveira et al. (2017) | Survey  (Brazil) | Brazilian postpartum women | 456  100% female | Revised Conflict Tactics Scale (CTS2) | Psychological violence, physical violence | **PTSD**  Posttraumatic Stress Disorder Checklist–Civilian Version  (PCL-C) |
| **59** | Peltzer and Pengpid (2017) | Survey  (Thailand) | Thai women | 207  100% female | Severity of Violence Against Women Scale” (SVAWS) and The Danger Assessment Scale (DAS) | Physical violence, sexual violence, psychological violence, danger | **Depression symptoms & suicidal behaviour**  Edinburgh Postnatal Depression Scale (EPDS), The Danger Assessment Scale (DAS) |
| **60** | Rogathi et al. (2017) | Cohort  (Tanzania) | Tanzanian pregnant women | 1013  100% female | WHO Multi-Country Study on Women Health and Domestic Violence Against Women | Emotional violence, physical violence, sexual, violence | **Depression during pregnant and postpartum**  Swahili version of The Edinburgh Postpartum Depression Scale (EPDS) |
| **61** | Wolford-Clevenger et al. (2017) | Survey  (USA) | US women seeking shelter | 134  100% female | Coercive Tactics subscale and the Revised Conflict Tactics Scale subscale | Physical violence, psychological violence | **Depression, PTSD, suicide ideation & suicide attempt**  Depression Anxiety Stress Scale, Post-Traumatic Stress Disorder Checklist – Civilian Version, Modified Scale for Suicidal Ideation, Lifetime-Suicide Attempt Self-Injury Count (L-SASI) |
| **62** | Bernstein et al. (2016) | Survey  (South Africa) | South African HIV+ pregnant women | 623  100% female | WHO Multi-Country Study on Women Health and Domestic Violence Against Women | Emotional violence, physical violence, sexual violence | **Depression, psychological distress, alcohol use & drug use,**  The Edinburgh Postnatal Depression Scale (EPDS), Kessler 10 scale (K-10), Alcohol Use Disorders Identification Test (AUDIT), Drug Use Disorders Identification Test (DUDIT) |
| **63** | DeCou et al. (2016) | Survey  (USA) | US treatment-seeking incarcerated women | 186  100% female | Severity of Violence Against Women Scale (SVAWS) | Physical violence, sexual violence | **PTSD & symptoms of dissociation**  PTSD Checklist–Civilian Version (PCL-C), Dissociative Experiences Scale–II (DES-II) |
| **64** | Ferrari et al. (2016) | Survey  (UK) | UK IPV help-seeking women | 260  100% female | Composite Abuse  Scale (CAS) | Emotional violence, physical violence, harassment | **Depression, anxiety, PTSD, & psychological distress symptoms**  Patient Health Questionnaire depression subscale (PHQ-9), Generalized Anxiety Disorder Scale (GAD-7), Weathers’ Posttraumatic Stress Disorder Check List, Clinical Outcomes in Routine Evaluation-Outcome Measure (CORE-OM) |
| **65** | Finnbogadottir et al. (2016) | Cohort  (Sweden) | Swedish pregnant women | 1939  100% female | NorVold Abuse Questionnaire (NorAQ) modified from the Abuse Assessment Screen (AAS) | Emotional violence, physical violence | **Depression & alcohol use**  The Edinburgh Postnatal Depression Scale (EPDS), Alcohol Use Disorders Identification Test (AUDIT) |
| **66** | Kastello et al. (2016) | Survey  (USA) | US pregnant women | 239  100% female | Revised Conflict Tactics Scale (CTS2) | Psychological violence, physical violence, sexual violence | **PTSD**  Davidson Trauma Scale (DTS) |
| **67** | Sanchez et al. (2016) | Survey  (Peru) | Peruvian pregnant women | 634  100% female | Adapted version of Demographic Health Survey Questionnaires and Modules: WHO Multi-Country Study on Violence Against Women | Physical violence, sexual violence | **Maternal antepartum depression & sleep disturbances**  Patient Health Questionnaire depression subscale (PHQ-9), Ford Insomnia Response to Stress Test (FIRST) |
| **68** | Tanimu et al. (2016) | Survey  (Nigeria) | Nigerian women | 393  100% female | Composite Abuse  Scale (CAS) | Physical violence, emotional violence, harassment, sexual violence | **Depression**  Patient Health Questionnaire-2 (PHQ-2) |
| **69** | Ziaei et al. (2016) | Survey  (Bangladesh) | Bangladeshi pregnant women | 1212  100% female | Modified version of Conflict Tactic Scale (CTS) | Physical violence, sexual violence, emotional violence, controlling behaviour | **Emotional distress**  Self-Report Questionnaire (SRQ-20) |
| **70** | Abdelhai and Mosleh (2015) | Survey  (Egypt) | Egyptian pregnant women | 376  100% female | Hurt, Insulted, Threaten, Scream inventory (HITS) | Physical violence, emotional/psychological violence | **Depression & anxiety**  Hospital Anxiety and Depression Scale (HADS) |
| **71** | Alhusen et al. (2015) | Survey  (USA) | US pregnant women | 166  100% female | Abuse Assessment Screen (AAS) | Exposure to IPV | **Depressive symptoms**  Edinburgh Postnatal Depression Scale (EPDS) |
| **72** | Barrios et al. (2015) | Survey  (Peru) | Peruvian pregnant women | 1521  100% female | Demographic Health Survey Questionnaires and Modules: WHO Multi-Country Study on Violence Against Women | Physical violence, sexual violence | **Depression symptoms**  Patient Health Questionnaire depression subscale (PHQ-9) |
| **73** | DeCou et al. (2015) | Survey  (USA) | US treatment-seeking incarcerated women | 102  100% female | Severity of Violence Against Women Scale (SVAWS) | Physical violence, sexual violence | **PTSD**  PTSD Checklist–Civilian Version (PCL-C) |
| **74** | Flanagan et al. (2015) | Cohort  (USA) | US pregnant women | 180  100% female | Revised Conflict Tactics Scale (CTS2) | Psychological violence, physical violence | **Depression and stress**  Center for Epidemiologic Studies Depression Scale (CES-D), Perceived Stress Scale (PSS) |
| **75** | Fonseca-Machado et al. (2015)a | Survey  (Brazil) | Brazilian pregnant women | 358  100% female | Brazilian version of instrument developed for WHO Multi-Country Study on Women’s Health and Domestic Violence | Psychological violence, physical violence, sexual violence | **Prenatal depression**  Edinburgh Postnatal Depression Scale (EPDS) |
| **76** | Fonseca-Machado et al. (2015)b | Survey  (Brazil) | Brazilian pregnant women | 358  100% female | Brazilian version of instrument developed for WHO Multi-Country Study on Women’s Health and Domestic Violence | Psychological violence, physical violence, sexual violence | **Anxiety & PTSD**  State-Trait Anxiety Inventory (STAI), Posttraumatic Stress Disorder Checklist - Civilian Version (PCL-C) |
| **77** | Hink et al. (2015) | Survey  (USA) | US female trauma patients | 81  100% female | Partner Violence Screen (PVS) and Woman Abuse Screening Test Short (WAST-Short) | Exposure to IPV | **Mental illness, alcohol & drug abuse**  MINI International Neuropsychiatric Interview version 5.0.0 (MINI 5.0.0), Alcohol Use Disorders Identification Test (AUDIT), Drug Abuse Screening Test (DAST-10) |
| **78** | Jackson et al. (2015) | Cohort  (USA) | US Mexican pregnant women | 320  100% female | The Pregnancy Risk Assessment Monitoring System (PRAMS), Center for Disease Control and Prevention | Physical violence, sexual violence, emotional violence | **Maternal depression symptoms & perceived stress**  Edinburgh Postnatal Depression Scale (EPDS), Revised Perceived Stress Scale (PSS) |
| **79** | Khadra et al. (2015) | Survey  (Lebanon) | Lebanese IPV women | 85  100% female | The Composite Abuse Scale (CAS) Physical Abuse subscale | Physical violence | **PTSD**  PTSD Checklist−Civilian Version (PCL-C) |
| **80** | McFarlane et al. (2015) | Survey  (USA) | US IPV women | 300  100% female | Severity of Violence Against Women Scale (SVAWS) and The Danger Assessment Scale (DAS) | Psychological violence, physical violence, sexual violence | **Depression, anxiety & PTSD**  Post-Traumatic Stress Disorder Scale, Brief Symptom Inventory (BSI) |
| **81** | Mertin et al. (2015) | Survey  (Australia) | Australian IPV women | 87  100% female | Adapted Conflicts Tactics Scale (ACTS) | Exposure to IPV | **Depression & anxiety**  Symptom Checklist 90 Revised (SCL-90-R) |
| **82** | Tirado-Munoz et al. (2015) | RCT  (Spain) | \| Spanish women receiving drug abuse treatment who reported IPV \| \| --- \| | 14  100% female | Composite Abuse  Scale (CAS) and Psychological Maltreatment of Women Inventory (PMWI) | Psychological violence, emotional violence, aggressiveness | **Depression**  Spanish version of the Beck Depression Inventory (BDI-II) |
| **83** | Tsai et al. (2015) | Cohort  (USA) | South African pregnant women | 1121  100% female | Conflict Tactics Scale (CTS) | Physical violence | **Depression**  Edinburgh Postnatal Depression Scale (EPDS) |
| **84** | Van Parys et al. (2015) | Survey  (Belgium) | Belgian pregnant women | 1894  100% female | Abuse Assessment Screen (AAS) and WHO Multi-Country Study on Women’s Health and Domestic Violence | Physical violence, sexual violence | **Depression, anxiety & stress**  Abbreviated Psychosocial Scale which included Centre for Epidemiologic Studies Depression Scale, the Speilberger Trait Anxiety Scale, and Schar Subjective Stress Scale |
| **85** | Agrawal et al. (2014) | Cohort  (USA) | US postnatal women | 734  100% female | Conflict Tactics Scale (CTS) | Emotional violence, physical violence, sexual violence | **Depression & stress**  Center for Epidemiologic Studies Depression Scale (CES-D) |
| **86** | Cort et al. (2014) | Cohort  (USA) | US IPV women | 32  100% female | Abusive Behaviour Inventory (ABI) | Physical violence, psychological violence | **Depression & PTSD**  Hamilton Rating Scale for Depression (HRSD), PHQ-9, 2001), Modified PTSD Symptom Scale (MPSS) |
| **87** | Flanagan et al. (2014) | Survey  (USA) | US IPV victim and perpetrators | 362  100% female | Psychological Maltreatment of Women Inventory-Short  version (PMSI-S); Revised Conflict Tactics Scales (CTS-2); Sexual Experiences Survey (SES) | Psychological violence, physical violence, sexual violence | **Depression, PTSD, alcohol & drug abuse**  Center for Epidemiologic Studies Depression Scale (CES-D), Posttraumatic Stress Diagnostic Scale (PDS), Alcohol Use Disorders Identification Test (AUDIT), Drug Abuse Screening Test (DAST) |
| **88** | Hellmuth et al. (2014) | Cohort  (USA) | US pregnant women | 180  100% female | Revised Conflict Tactics Scale (CTS2) | Psychological violence, physical violence | **Depression, alcohol misuse & stress**  Center for Epidemiologic Studies Depression Scale (CES-D), Alcohol Use Disorders Identification Test (AUDIT), Perceived Stress Scale (PSS) |
| **89** | Kabir et al. (2014) | Survey  (Bangladesh) | Bangladeshi postpartum women | 660  100% female | WHO Multi-Country Study on Women’s Health and Domestic Violence | Physical violence, sexual violence, emotional violence | **Maternal depressive symptoms**  Edinburgh Postnatal Depression Scale (EPDS) |
| **90** | Kelly and Pich (2014) | Cohort  (USA) | US IPV women | 22  100% female | Index of Spouse Abuse (ISA) | Physical violence, non-physical violence | **Depression & PTSD**  Center for Epidemiologic Studies Depression Scale (CES-D), PTSD Checklist–Civilian Version (PCL-C) |
| **91** | Lara Ma et al. (2014) | Survey  (Mexico) | Mexican pregnant women | 250  100% female | Intimate Partner Violence Evaluation Scale | Physical violence, verbal violence | **Depression**  Center for Epidemiologic Studies Depression Scale (CES-D) |
| **92** | Ludermir et al. (2014) | Survey  (Brazil) | Brazilian pregnant women | 1120  100% female | WHO Multi-Country Study on Women’s Health and Domestic Violence | Psychological violence, physical violence, sexual violence, controlling behaviour | **Common mental disorders**  Self-Reporting Questionnaire (SRQ-20) |
| **93** | McFarlane et al. (2014) | Cohort  (USA) | US IPV women | 46  100% female | Severity of Violence Against Women Scale (SVAWS) and The Danger Assessment Scale (DAS) | Psychological violence, physical violence, sexual violence | **PTSD & psychological distress**  Post-Traumatic Stress Disorder (PTSD) Scale, Brief Symptom Inventory (BSI) |
| **94** | Nongrum et al. (2014) | Cohort  (India) | Indian pregnant women | 150  100% female | Abuse Assessment Screen (AAS) | Exposed to IPV  Non-exposed to IPV | **Perinatal depression**  Edinburgh Postnatal Depression Scale (EPDS) |
| **95** | Reichenheim et al. (2014) | Survey  (Brazil) | Brazilian postpartum women | 810  100% female | Brazilian version of the Revised Conflict Tactics Scale (CTS2) | Psychological violence, physical violence | **Common mental disorders**  Self-Reporting Questionnaire (SRQ-20) |
| **96** | Symes et al. (2014) | Survey  (USA) | US women | 300  100% female | Severity of Violence Against Women Scale (SAVAWS) | Sexual violence, physical violence, risk of lethality | **Anxiety & PTSD**  Post-Traumatic Stress Disorder Scale (PTSD), Brief Symptom Inventory |
| **97** | Almeida et al. (2013) | Survey  (Portugal) | Portuguese pregnant women | 184  100% female | Conflict Tactics Scale (CTS) | Psychological violence, physical violence, sexual violence | **Depression & psychological symptoms**  Inventory of the Clinical Evaluation of Depression (IACLIDE), Brief Symptom Inventory (BSI) |
| **98** | Ayub et al. (2013) | Survey  (Pakistan) | Pakistan women | 640  100% female | Women’s Experience with Battering (WEB) | Verbal violence, physical violence | **PTSD & suicide ideation**  Mini-International Neuropsychiatric Interview (MINI) |
| **99** | Dennis and Vigod (2013) | Cohort  (Canada) | Canadian pregnant women | 166  100% female | Assessment of Interpersonal Violence and Substance Use (ALPHA) | Physical violence, psychological violence, sexual violence, child abuse | **Depressive symptoms**  Edinburgh Postnatal Depression Scale (EPDS) |
| **100** | Edmond et al. (2013) | Cohort  (USA) | US IPV women | 50  100% female | Composite Abuse Scale (CAS) | Physical violence, emotional abuse, harassment | **Depression, PTSD & alcohol abuse**  Diagnostic Interview Schedule IV (DIS-IV) |
| **101** | Faisal-Cury et al. (2013) | Survey  (Brazil) | Brazilian pregnant women | 701  100% female | WHO Multi-Country Study on Women’s Health and Domestic Violence | Psychological violence, physical violence, sexual violence | **Postpartum depression**  Self-Report Questionnaire (SRQ-20) |
| **102** | Fisher et al. (2013) | Cohort  (Vietnam) | Vietnamese pregnant women | 497  100% female | WHO Multi-Country Study on Women’s Health and Domestic Violence | Physical violence, sexual violence, emotional violence | **Postnatal depression & suicidal thoughts**  Edinburgh Postnatal Depression Scale – Vietnam Validation (EPDS) |
| **103** | Illangasekare et al. (2013) | Survey  (USA) | US IPV women | 96  100% female | Revised Conflict Tactics Scale (CTS2) | Physical violence, sexual violence, psychological violence | **Depression**  Center for Epidemiologic Studies Depression Scale (CES-D) |
| **104** | Jaquier et al. (2013) | Survey  (USA) | US IPV women | 212  100% female | Conflict Tactics Scales-2 (CTS-2); Sexual Experiences Survey (SES) and the Psychological Maltreatment of Women Inventory (PMWI) | Physical violence, sexual violence, psychological violence | **Depression, PTSD & self-harm**  Center for Epidemiologic Studies Depression Scale (CES-D), Posttraumatic Stress Diagnostic Scale (PDS), Deliberate Self-harm Inventory (DSHI) |
| **105** | Kamimura et al. (2013) | Survey  (USA) | US help-seeking women from Family Justice Centres | 117  100% female | Danger Assessment | Danger assessment | **Depression, psychological distress & mental health**  Patient Health Questionnaire -9 (PHQ-9), General Health Questionnaire (GHQ),  Short Form (SF)-12 |
| **106** | Mahenge et al. (2013) | Survey  (Tanzania) | Tanzanian pregnant women | 1180  100% female | Conflict Tactics Scale (CTS) | Physical violence, sexual violence | **Depression, anxiety & PTSD**  John Hopkins Symptoms checklist with 25 questions (HSCL-25), Posttraumatic Diagnostic Scale (PDS) |
| **107** | Mapayi et al. (2013) | Survey  (Nigeria) | Nigerian women | 373  100% female | Composite Abuse Scale (CAS) | Exposure to IPV | **Depression & anxiety**  Hospital Anxiety and Depression Scale |
| **108** | Ogbonnaya et al. (2013) | Cohort  (USA) | US pregnant women | 76  100% female | Conflict Tactics Scale 2 (CTS2) | Physical violence | **Depressive symptoms**  Center for Epidemiologic Studies Depression Scale (CES-D) |
| **109** | Peterson et al. (2013) | Survey  (USA) | US IPV women | 42  100% female | Danger Assessment (DA) and Index of Spouse Abuse (ISA) | Danger assessment | **Depression & PTSD**  Beck Depression Inventory, Second Edition (BDI-II), Posttraumatic Stress Diagnostic Scale (PDS) |
| **110** | Sabri et al. (2013) | Survey  (USA) | US African-American and African-Caribbean women | 431  100% female | Severity of Violence against Women Scale (SVAWS), Women’s Experiences of Battering (WEB), Danger Assessment (DA) instrument | Physical violence, sexual violence, psychological violence, | **Depression & PTSD**  Center for Epidemiologic Studies Depression Scale (CES-D-10), Primary Care Post-Traumatic Stress Disorder Screening (PC-PTSD) |
| **111** | Saito at al. (2013) | Survey  (Thailand) | Thai pregnant women | 421  100% female | Psychological Maltreatment of Women Inventory-Short Form (PMWI—SF), and the Severity of Violence Against Women scale (SVAW) | Physical violence | **Mental health**  Short Form 12-Item Health Survey (SF-12) |
| **112** | Wong et al. (2013) | RCT  (Hong Kong) | Hong Kong IPV women | 197  100% female | The Chinese version of the Abuse Assessment Screen (C-AAS), Revised Conflict Tactics Scales (CTS2) | Psychological violence, physical violence, sexual coercion | **Depressive symptoms**  The Chinese version of the Beck Depression Inventory Version II (C-BDI-II) |
| **113** | Young-Wolff et al. (2013) | Survey  (USA) | US IPV women | 412  100% female | The Psychological Maltreatment of Women Inventory-Short version (PMWI-S), The Sexual Experiences Survey (SES), The Conflict Tactics Scale-2 (CTS-2), Past Abusive Behavior Inventory | Physical violence, sexual violence, psychological violence | **Depression, PTSD, alcohol & drug problems**  Center for Epidemiologic Studies Depression Scale (CES-D), Posttraumatic Diagnostic Scale (PDS), Alcohol Use Disorders Identification Test (AUDIT), Drug Abuse Screening Test (DAST) |
| **114** | Zakar et al. (2013) | Survey  (Pakistan) | Pakistani women | 373  100% female | Revised Conflict Tactics Scale (CTS2) | Psychological violence, physical violence | **Poor mental health**  Self-Reporting Questionnaire (SRQ-20) |
| **115** | Al-Modallal (2012) | Survey  (Jordan) | Palestinian help-seeking women in refugee camps | 267  100% female | WHO Multi-Country Study on Women’s Health and Domestic Violence | Controlling behaviour/psychological violence | **Depression, anxiety & stress**  Center for Epidemiologic Studies Depression Scale (CES-D), Depression Anxiety Stress Scales |
| **116** | Budhathoki et al. (2012) | Cohort  (Nepal) | Nepalese pregnant women | 72  100% female | WHO Multi-Country Study on Women’s Health and Domestic Violence | Psychological violence, physical violence, sexual violence | **Postpartum depression**  Edinburgh Postnatal Depression Scale (EPDS) |
| **117** | Comeau and Davies (2012) | Survey  (Canada) | Canadian IPV women | 309  100% female | A modified version of the Index of Spouse Abuse (ISA) | Physical and non-physical violence | **Depression**  Center for Epidemiologic Studies Depression Scale (CES-D) |
| **118** | Fleming et al. (2012) | Survey  (USA) | US IPV women | 192  100% female | Conflict Tactics Scale (CTS2) | Physical violence, sexual coercion, psychological violence, injury | **PTSD**  PTSD Checklist—Civilian |
| **118** | Gerber et al. (2012) | Survey  (USA) | US seeking-treatment for headache from HC | 92  100% female | STaT (slapped, threatened and throw)  Partner Violence Screen (PVS) | Lifetime IPV | **PTSD**  Seven-item modified Breslau screening tool for PTSD |
| **120** | Gilchrist et al. (2012) | Survey  (Spain) | Spanish drug user women in treatment | 118  100% female | Composite Abuse Scale (CAS) | Exposure to IPV | **Major depression, PTSD, self-harm, suicide attempts, borderline personality & substance use disorders (SUD)**  Spanish Psychiatric Research Interview for Substance and Mental Disorders (PRISM) |
| **121** | Groves et al. (2012) | Survey  (South Africa) | South African pregnant women | 1500  100% female | WHO Multi-Country Study on Women’s Health and Domestic Violence | Physical violence, Psychological violence, Sexual violence | **Emotional distress**  Hopkins Symptom Checklist (HSCL-25) |
| **122** | Holden et al. (2012) | Survey  (USA) | US pregnant women | 602  100% female | Woman Abuse Screening Tool (WAST) | Exposure to IPV | **Depression, alcohol abuse & drug abuse**  Alcohol Use Disorders Identification Test (AUDIT), Drug Abuse Screening Test (DAST), Edinburgh Postnatal Depression Scale (EPDS) |
| **123** | Illangasekare et al. (2012) | Survey  (USA) | US HIV+ women receiving care in HIV clinics | 196  100% female | Partner Violence Screen | Exposure to IPV | **Depression**  Center for Epidemiologic Studies Depression Scale (CES-D) |
| **124** | Lobato et al. (2012) | Survey  (Brazil) | Brazilian postpartum mothers | 811  100% female | Revised Conflict Tactics Scale (CTS2) | Physical violence | **Postpartum depression symptoms, alcohol & drug misuse**  Edinburgh Postnatal Depression Scale (EPDS), TWEAK, CAGE |
| **125** | Matseke et al. (2012) | Survey  (South Africa) | HIV-positive and HIV-negative pregnant women | 1502  100% female | Revised Conflict Tactics Scale (CTS2) | Physical violence, sexual violence, emotional violence | **Psychological distress**  Kessler Psychological Distress Scale (K-10) |
| **126** | Miszkurka et al. (2012) | Survey  (Canada) | Canadian pregnant women | 5162  100% female | Abuse Assessment Screen (AAS) | Physical violence, Threats, Armed threats, Sexual violence | **Depression**  Center for Epidemiologic Studies Depression Scale (CES-D) |
| **127** | Nathanson et al. (2012) | Survey  (USA) | US IPV women | 101  100% female | Revised Conflict Tactics Scale (CTS2) | Psychological violence, physical violence, sexual coercion, injury victimization | **Depression, alcohol and drug abuse & PTSD**  Structured Clinical Interview for DSM-IV (SCID), Clinician Administered Posttraumatic Stress Disorder Scale (CAPS) |
| **128** | Norwood and Murphy (2012) | Survey  (USA) | US partners of IPV perpetrators seeking counselling for perpetration | 216  100% female | Revised Conflict Tactics Scale (CTS2), Multidimensional measure of emotional abuse (MMEA), Sexual Experiences Survey (SES) | Physical violence, sexual coercion, emotional violence, sexual violence | **PTSD**  PTSD Checklist–Civilian Version (PCL-C) |
| **129** | Quelopana (2012) | Survey  (Chile) | Chilean pregnant women | 163  100% female | Women Abuse Screen Tool (WAS) | Psychological violence, physical violence, sexual violence | **Postpartum depressive symptoms**  Postpartum Depression Screening Scale (PDSS) |
| **130** | Saito et al. (2012) | Survey  (Thailand) | Thai post-partum women | 274  100% female | Psychological Maltreatment of Women Inventory-Short Form (PMWI—SF), and the Severity of Violence Against Women scale (SVAW) | Psychological violence, physical violence, sexual violence | **Depressive symptoms**  Short Form 12-Item Health Survey (SF-12) |
| **131** | Sullivan et al. (2012) | Survey  (USA) | US substance abuse IPV exposed women | 143  100% female | Conflict Tactics Scale-2 (CTS-2), Sexual Experiences Survey (SES)  Psychological Maltreatment of Women Inventory (PMWI) | Psychological violence, physical violence, sexual violence | **Depression, PTSD & alcohol problems**  Center for Epidemiologic Studies Depression Scale (CES-D), Posttraumatic Stress Diagnostic Scale (PDS), Alcohol Use Disorders Identification Test (AUDIT) |
| **132** | Woolhouse et al. (2012) | Cohort  (Australia) | Australian pregnant women | 1507  100% female | Short version of the Composite Abuse Scale (CAS) | Emotional violence, physical violence | **Postnatal depression**  Edinburgh Postnatal Depression Scale (EPDS) |
| **133** | Zacarias et al. (2012) | Survey  (Mozambique) | Mozambique IPV women | 1442  100% female | Conflict Tactic Scales-Version 2 (CTS2), Controlling Behaviors Scale – Revised (CBS) | Psychological violence, Physical violence, Sexual violence, Injury | **Depression & anxiety**  Symptom Check List- Revised (SCL-90-R) |
| **134** | Zou et al. (2012) | Survey  (China) | Chinese pregnant women | 846  100% female | Chinese version of the Abuse Assessment Screen (AAS) | Emotional violence, physical violence, sexual violence | **Postnatal depression**  Edinburgh Postnatal Depression Scale (EPDS) |
| **Studies adopting community sample (135- 201) (household survey, large populations survey, students, community-based studies)** | | | | | | | |
| **135** | Ahmadabadi et al.  (2020) | Cohort  (Australia) | University students | 1528  100% female | Modified version of the Composite Abuse Scale (CAS) | Physical violence, emotional violence, harassment | **Depression & anxiety**  Composite International  Diagnostic Interview (CIDI) |
| **136** | Aye et al,  (2020) | Survey  (Burma) | Myanmar national sample | 2383  49.6% female  50.4% male | Modified version of the Conflict Tactics Scale (CTS) | Physical violence, sexual violence, emotional violence | **Mental distress**  Hopkins Symptom Checklist-10  (HSCL-10) |
| **137** | Bedford et al. (2020) | Survey  (China) | Chinese low-income women | 156  100% female | Chinese Abuse Assessment Screen (C-AAS) | Emotional violence, physical violence | **Depression, anxiety & stress**  Depression Anxiety Stress Scale (DASS-21) |
| **138** | Brar et al. (2020) | Survey  (Malawi) | Malawian adolescent girls | 995  100% female | Conflict Tactics Scale (CTS) | Emotional violence, physical violence, sexual violence, controlling behaviour | **Depression**  Center for Epidemiologic Studies Depression Scale (CES-D-10) |
| **139** | Brown et al. (2020) | Cohort  (Australia) | Australian women | 1507  100% female | Composite Abuse Scale (CAS) | Emotional violence, physical violence, sexual violence | **Symptoms of depression, anxiety, PTSD, & depressive symptoms during pregnancy**  Center for Epidemiologic Studies Depression Scale (CES-D), Beck Anxiety Inventory (BAI), Post-Traumatic Stress Disorder Checklist–Civilian version (PCL-C), Edinburgh Postnatal Depression Scale (EPDS) |
| **140** | Dardis et al.  (2020) | Cohort  (USA) | US college students | 1268  68.5% female  31.5% male | Conflict Tactics Scale –short version | Sexual assault; intimate partner violence; psychological/verbal violence | **Depressive symptoms and PTSD symptoms**  Center for Epidemiologic Studies Depression Scale (CES-D), PTSD Checklist for Diagnostic and Statistical Manual of Mental Disorders, Fifth Edition (DSM-5) |
| **141** | Falb et al. (2020) | Survey  (Syria) | Syrian married women | 214  100% female | WHO Multi-Country Study on Women’s Health and Domestic Violence | Physical violence, sexual violence, emotional violence | **Depression**  Patient Health Questionnaire (PHQ-9) |
| **142** | Morris et al. (2020) | Survey  (Kenya) | Kenyan women | 873  100% female | Adapted versions of the Abuse Assessment Screen (AAS) and Partner Violence Screen (PVS) | Emotional violence, physical violence, sexual violence | **Depression**  Patient Health Questionnaire Depression Scale (PHQ-8) |
| **143** | Mugoya et al. (2020) | Survey  (USA) | African American low-income women | 664  100% female | Select items from the Revised Conflict Tactics Scale (CTS2) | Physical violence, psychological violence | **Depression symptoms**  Center for Epidemiologic Studies Depression Scale (CES-D) |
| **144** | Richardson et al. (2020) | RCT  (India) | Indian women | 3010  100% female | Demographic and Health Survey’s (DHS) Domestic Violence Module, which were adopted from the Conflict Tactics Scale (CTS) | Controlling behaviour, psychological violence, physical violence | **Symptoms of mental distress**  General Health Questionnaire (GHQ-12) translated into Hindi |
| **145** | Sezgin and Punamaki (2020) | Survey  (Turkey) | Turkish women | 1569  100% female | Revised Conflict Tactics  Scale (CTS-2) | Physical violence, psychological violence, assault, sexual coercion, emotional neglect | **PTSD symptoms & psychiatric distress**  National Stressful Events Survey PTSD Short Scale (NSESSS-PTSD), General Health Questionnaire (GHQ-28) |
| **146** | Stein et al. (2020) | Survey  (USA) | US Latinas | 77  100% female | Revised Conflict Tactics  Scale (CTS-2) | IPV exposure | **Depressive symptoms & PTSD**  Center for Epidemiologic Studies Depression Scale (CES-D), Posttraumatic Diagnostic Scale (PDS) |
| **147** | Winter et al. (2020) | Survey  (Kenya) | Women in informal settlements Kenya | 361  100% female | Modified version of the domestic violence module Demographic and Health Surveys (DHS) | Psychological violence, sexual violence, physical violence | **Mental health, psychological distress, MDD & alcohol use**  Patient Health Questionnaire-9 Depression Scale (PHQ-9), Swahili version of the Kessler Scale of Psychological Distress (K10), 36-Item Short Form Health Survey (SF-36) |
| **148** | Xu et al. (2020) | Survey  (China) | Chinese men/ women | 2268  52% female  48% male | WHO Multi-Country Study on Women’s Health and Domestic Violence | Controlling behaviour (personal and financial), Physical violence, Psychological violence, Sexual violence | **Depressive symptoms**  Center for Epidemiologic Studies Depression Scale (CES-D) |
| **149** | An et al. (2019) | Survey  (Korea) | Korean women | 3160  100% female | Massachusetts Youth Risk Behavior Survey | Physical violence, sexual violence | **MDD, anxiety, PTSD & alcohol abuse**  Korean version of the CIDI2.1 (K-CIDI 2.1) (WHO-Composite International Diagnostic Interview (K-CID) |
| **150** | Esie et al. (2019) | Cohort  (Bangladesh) | Bangladeshi married women | 3290  100% female | Revised Conflict Tactics Scale (CTS) and WHO Multi-Country Study on Women’s Health and Domestic Violence | Psychological violence, physical violence, sexual violence and injury | **Major depressive episodes**  Edinburgh Postnatal  Depression Scale (EPDS) |
| **151** | Han et al. (2019) | Cohort  (Korea) | Korean married adults | 9217  51% female  49% male | Conflict Tactics Scale (CTS) | Verbal violence, physical violence | **Depressive symptoms**  Center for Epidemiologic Studies Depression (CES-D-11) Scale |
| **152** | Lysova et al. (2019) | Survey  (Canada) | Canada general population | 14,575  49% female  51% male | Revised Conflict Tactics Scale—Victimization (CTS2) | Physical violence, sexual violence | **PTSD symptoms**  Primary Care PTSD Screen for DSM-5 |
| **153** | Nyato et al. (2019) | Survey  (Tanzania) | Adolescent and young women | 3013  100% female | WHO Multi-Country Study on Women’s Health and Domestic Violence | Sexual violence, emotional violence, physical violence | **Depression symptoms**  Patient Health Questionnaire (PHQ-4) |
| **154** | Ross et al. (2019) | Survey  (USA) | US college students | 885  66% female  34% male | SCIRS Sexual Coercion and Severity of Violence Against Women Scale (SVAWS) | Sexual violence, sexual coercion | **Depression & anxiety symptoms**  The Hopkins Symptom Checklist (HSCL) |
| **155** | Voth Schrag et al. (2019) | Survey  (USA) | US female college students | 435  100% female | The Abusive Behavior Inventory (revised) (ABI(R)), The Scale of Economic Abuse (SEA) | Economic abuse, physical violence, sexual violence | **Depression & PTSD symptoms**  Center for Epidemiologic Studies Depression (CES-D), the PTSD Checklist for DSM-5 (PCL-5) |
| **156** | Wong et al. (2019) | Survey  (Hong Kong) | Chinese college students | 1015  58.5% female  41.5% male | The sexual abuse subscale of the Chinese Revised Conflict Tactic Scale (CTS-2) | Sexual coercion, sexual violence | **Anxiety & depression**  Chinese version of the Hospital Anxiety and Depression Scale (HADS) |
| **157** | Wright et al. (2019) | Survey  (USA) | US women aged 24-32 | 7392  100% female | Revised Conflict Tactics Scale (CTS2) | Physical violence, sexual violence | **Depression & alcohol dependence**  Center for Epidemiologic Studies Depression (CES-D), Alcohol Dependence  measure from the DSM-IV |
| **158** | Yuan and Hesketh (2019) | Survey  (China) | Chinese women | 2987  100% female | Revised Conflict Tactics Scale (CTS2) and the Composite Abuse Scale (CAS) | Psychological violence, physical violence, sexual violence | **Depression**  Center for Epidemiologic Studies Depression (CES-D) |
| **159** | Alangea et al. (2018) | Survey  (Ghana) | Ghanian women | 2000  100% female | WHO Multi-Country Study on Women’s Health and Domestic Violence | Sexual violence, physical violence, economic violence, emotional violence | **Depression**  Center for Epidemiologic Studies Depression (CES-D) |
| **160** | Chang et al. (2018) | Survey  (USA) | US men and women | 101  71% female  29% male | Hurt, Insulted, Threatened With Harm, and Screamed at Them Scale (HITS) | Exposure to domestic partner violence | **Suicidal behaviours**  Suicidal Behaviors Questionnaire–Revised (SBQ-R) |
| **161** | Gibbs et al. (2018) | Survey  (South Africa) | South African women | 680  100% female | Adapted version of the WHO MCS survey, the UN Multi-Country Study for women (UNMCS) | Physical violence, sexual violence | **Depression & suicidal ideation**  Center for Epidemiologic Studies Depression (CES-D) |
| **162** | Indu et al. (2018) | Survey  (India) | South Indian women | 60  100% female | Domestic Violence Questionnaire (DVQ) | Physical violence, sexual violence | **Depression & anxiety**  MINI (Mini International Neuropsychiatric Interview) Malayalam Version |
| **163** | Kavak et al. (2018) | Survey  (Turkey) | Turkish women | 1025  100% female | Domestic Violence Scale (DVS) | Emotional violence, verbal violence, physical violence, sexual violence, financial control | **Probability of suicide**  Suicide probability scale |
| **164** | Maru et al. (2018) | Survey  (USA) | Asian American women | 173  100% female | Revised Conflict Tactics Scale (CTS2) | Psychological violence, sexual coercion, physical violence | **Lifetime suicidal ideation/intent**  Columbia Suicide Severity Rating Scale (C-SSRS) |
| **165** | Mathur et al. (2018) | Survey  (Kenya and Zambia) | Kenyan and Zambian young women | 3693  100% female | WHO Multi-Country Study on Women’s Health and Domestic Violence | Sexual violence | **Depression & anxiety**  PHQ-4 mental health scale |
| **166** | Qin and Yan (2018) | Survey  (China) | Older Chinese | 453  56.3% female  43.7% male | Potentially Harmful Behavior (PHB) scale adapted from WHO Multi-Country Study on Women’s Health and Domestic Violence | Physical violence, verbal violence, psychological violence | **Poor mental health**  General Health Questionnaire (GHQ) |
| **167** | Ridings et al. (2018) | Cohort  (USA) | US female caregivers | 548  100% female | Revised Conflict Tactics Scale (CTS2) | Exposure to IPV | **Depression**  Beck Depression Inventory–2nd Edition (BDI-II) |
| **168** | Ruiz-Perez et al. (2018) | Survey  (Spain) | Spanish general population | 4507  51% female  49% male | WHO Multi-Country Study on Women’s Health and Domestic Violence | Physical violence, emotional violence, sexual violence | **Depression, mood disorders, PTSD, alcohol and substance abuse, suicidal ideation, & personality disorders**  SF-12 questionnaire, MINI International  Neuropsychiatric Interview |
| **169** | Shah et al. (2018) | Survey  (USA) | US general population | 1615  58% female  42% male | National Intimate Partner and Sexual Violence Survey | Threat, physical violence, sexual violence | **Psychotic experiences**  World Health Organization (WHO) Composite International Diagnostic Interview (CIDI) psychosis screen |
| **170** | Simmons et al. (2018) | Cohort  (USA) | US general population | 879  53% female  47% male | Conflict Tactics Scale (CTS) | IPV Victimization  IPV Perpetration | **Depression**  Diagnostic Interview Schedule (DIS) for DSM-IV |
| **171** | Yalch and Levendosky (2018) | Survey  (USA) | US young adult women | 654  100% female | Revised Conflict Tactics Scale (CTS2) | Exposure to IPV | **Hazardous alcohol use**  Alcohol Use Disorder Identification Test (AUDIT) |
| **172** | Gehring and Vaske (2017) | Cohort  (USA) | US adolescent | 9863  52.4% female  47.6% male | Conflict Tactics Scale (CTS) | Exposure to IPV | **Depressive symptoms & alcohol related problems**  Center for Epidemiologic Studies Depression (CES-D), Diagnostic and Statistical Manual of Mental Disorders |
| **173** | Kapiga et al. (2017) | Survey  (Tanzania) | Tanzanian women | 1021  100% female | WHO Multi-Country Study on Women’s Health and Domestic Violence | Physical violence, sexual violence, controlling behaviour | **Poor mental health**  Self-Report Questionnaire (SRQ-20) |
| **174** | Liu et al. (2017) | Survey  (USA) | Multi countries general population | 34676  44.6% female  55.4% male | WHO World Mental Health (WMH) surveys | Physical violence (experience as a child), sexual violence (by romantic partner) | **PTSD**  Composite International Diagnostic Interview (CIDI) |
| **175** | Mendonca et al. (2017) | Cohort  (Brazil) | Brazilian women | 390  100% female | WHO Multi-Country Study on Women Health and Domestic Violence Against Women | Psychological violence, physical violence, sexual violence | **Mental health**  Self-Report Questionnaire (SRQ-20) |
| **176** | Yalch et al. (2017) | Survey  (USA) | US female students | 654  100% female | Revised Conflict Tactics Scale (CTS2) | Physical violence, sexual violence, psychological violence | **Trauma symptoms**  Trauma Symptom Checklist (TSC-33) |
| **177** | Ally et al. (2016) | Survey  (Brazil) | Brazilian men and women | 3007  64% female  36% male | Revised Conflict Tactics Scale (CTS2) | Victimisation, perpetration | **Alcohol disorders**  Brazilian version of the Composite International Diagnostic Interview (DSM-5) |
| **178** | Al-Modallal (2016) | Survey  (Jordan) | Jordanian college female students | 97  100% female | The Safe Dates–Physical Violence Victimization Scale | Physical violence, sexual violence | **Depressive symptoms & stress**  Center for Epidemiologic Studies Depression (CES-D), Depression Anxiety Stress Scales |
| **179** | De Barros et al. (2016) | Survey  (Brazil) | Brazilian women | 245  100% female | WHO Multi-Country Study on Women Health and Domestic Violence Against Women | Emotional violence, physical violence, sexual violence | **Common mental disorders**  Self-Reporting Questionnaire (SRQ-20) |
| **180** | Ulloa et al. (2016) | Survey  (USA) | US general population | 7187  60% female  40% male | Modified version of the Conflicts Tactics Scale (CTS) | No violence, unidirectional violence, bidirectional violence | **Depression**  Center for Epidemiologic Studies Depression Scale (CES-D) |
| **181** | Hellemans et al. (2015a) | Survey  (Belgium) | Turkish population in Belgium | 392  50.2% female  49.8% male | Modified version of the Conflicts Tactics Scale (CTS), WHO Multi-Country Study on Women Health and Domestic Violence Against Women | Physical violence, psychological violence | **Mental health**  Mental Health Inventory (MHI-5) |
| **182** | Hellemans et al. (2015b) | Survey  (Belgium) | Flanders general population | 1448  48% female  52% male | Modified version of the Conflicts Tactics Scale (CTS), WHO Multi-Country Study on Women Health and Domestic Violence Against Women | Physical violence, psychological violence | **Mental health**  Mental Health Inventory (MHI-5) |
| **183** | Kulwicki et al. (2015) | Survey  (USA) | Arab US women | 312  100% female | Danger Assessment Scale (DAS) | Physical violence, threats, psychological | **Depressive symptoms**  Center for Epidemiologic Studies Depression Scale (CES-D) |
| **184** | La Flair et al.  (2015) | Cohort  (USA) | US female healthcare workers | 215  100% female | Modified version of the Abuse Assessment Screen (AAS) | Physical violence, sexual violence, emotional violence | **Depression & PTSD**  Center for Epidemiologic Studies Depression Scale short form (CESD-10), PTSD screen (PC-PTSD) |
| **185** | Ouellet-Morin et al. (2015) | Cohort  (UK) | UK mothers | 978  100% female | Conflict Tactics Scale (CTS) | Exposure to IPV | **Depressive disorders & psychosis spectrum symptoms**  Diagnostic Interview Schedule (DIS) for DSM-IV, Michigan Alcoholism Screening Test, Drug Abuse Screening Test, Psychosis Screening Questionnaire (PSQ) |
| **186** | Svavarsdottir et al. (2015) | Survey  (Iceland) | Icelandic women | 306  100% female | Woman Abuse Screening Test (WAST) | Overall score reported | **PTSD**  Primary Care Post-Traumatic Stress Disorder (PC-PTSD) Screening Tool |
| **187** | Wright et al. (2015) | Cohort  (USA) | US female caregivers | 2959  100% female | Conflict Tactics Scale (CTS) | Physical violence, threatened | **Depression symptoms**  Composite International Diagnostic Interview (UM-CIDI) |
| **188** | Decker et al. (2014) | Survey  (Multi states: USA, India, Nigeria, South Africa, China) | Adolescent females from 5 countries | 1112  100% female | Conflict Tactics Scale (CTS2) | Physical violence, sexual violence, threatened | **Depression symptoms & suicidal ideation**  Center for Epidemiologic Studies Depression Scale (CES-D) |
| **189** | Jeter et al. (2014) | Survey  (USA) | US female students | 232  100% female | Revised Conflict Tactics Scale (CTS2), Measure of Psychologically Abusive Behaviors | Psychological violence, physical violence | **PTSD symptoms**  Posttraumatic Stress Disorder Checklist |
| **190** | Renner et al. (2014) | Survey  (USA) | US rural couples | 548  50% female  50% male | Conflict Tactics Scale (CTS) | Physical violence, emotional violence | **Depressive symptoms**  Center for Epidemiologic Studies Depression Scale |
| **191** | Tiwari et al. (2014) | Survey  (Hong Kong) | Chinese women | 745  100% female | Chinese Version of the Abuse Assessment Screen (C-AAS); Chinese version of the Revised Conflict Tactics Scale (C-CTS2); The Chinese version of the Revised Controlling Behaviors Scale (C-CBS-R). | Physical violence, sexual violence | **Depressive & PTSD symptoms**  Chinese version of the 21-item Beck Depression Inventory version II (C-BDI-II) |
| **192** | Watkins et al. (2014) | Cohort  (USA) | US young women | 375  100% female | Revised Conflict Tactics Scale (CTS2) | Physical victimization, psychological victimization, physical IPA, psychological IPA | **Depressive symptoms**  Depression Subscale of the Depression Anxiety Stress Scales (DASS-21) |
| **193** | Adams et al. (2013) | Cohort  (USA) | US low-income women | 503  100% female | Conflict Tactics Scale (CTS) | Physical violence, sexual violence, emotional violence | **Major depression & anxiety disorder**  WHO-CIDI-SF |
| **194** | Kim et al. (2013) | Survey  (Korea) | Korean national sample | 3153  100% female | Conflict Tactics Scale (CTS) | Physical violence | **Depression**  Center for Epidemiologic Studies Depression Scale (CES-D) |
| **195** | Meekers et al. (2013) | Survey  (Bolivia) | Bolivian women | 10,119  100% female | Bolivia Demographic and Health Survey [based on the Conflict Tactics Scale (CTS)] | Psychological violence, physical violence, sexual violence | **Depression, anxiety & symptoms of psychotic disorders**  Self-Reporting Questionnaire 20 (SRQ-20), Self-Reported Questionnaire (SRQ-25) |
| **196** | Verduin et al. (2013) | Survey  (Rwanda) | Rwandan general population | 241  46.1% female  53.9% male | Revised Conflict Tactics Scale, Short Version (CTS2S) | IPV victims  IPV perpetrators | **Common mental disorders & suicidal ideation**  Self-Reporting Questionnaire—20 Items (SRQ-20) |
| **197** | Jina et al. (2012) | Survey  (South Africa) | South African young women | 1293  100% female | WHO Multi-Country Study on Women Health and Domestic Violence Against Women | Emotional violence, physical violence, sexual violence | **Depression & alcohol use**  Center for Epidemiologic Studies Depression Scale (CES-D), Alcohol Use Disorder Identification Test (AUDIT) |
| **198** | Kaplan et al. (2012) | Cohort  (USA) | US low-income women | 2269  100% female | Revised Conflict Tactics Scale (CTS2) | Physical violence, sexual coercion, psychological violence | **Psychological distress**  Brief Symptom Inventory (BSI-18) |
| **199** | La Flair et al. (2012) | Cohort  (USA) | US female healthcare workers | 1438  100% female | Abuse Assessment Screen (AAS) | Sexual violence, physical violence, psychological violence, stalking | **Depressive symptoms**  Center for Epidemiologic Studies Depression Scale (CES-D) |
| **200** | Lucea et al. (2012) | Case-control  (Baltimore, MD, USA and St. Croix and St. Thomas, U.S. Virgin Islands (USVI)) | US African and African Caribbean women | 781  100% female | Abuse Assessment Screen (AAS), Women’s Experiences of Abuse (WEB); Severity of Violence Against Women instrument (SVAWS) and Danger Assessment Scale (DAS) | Emotional violence, physical violence, sexual violence | **Depressive symptoms & PTSD**  Center for Epidemiologic Studies Depression Scale (CES-D), Primary Care Post Traumatic Stress Disorder Screening (PC-PTSD) |
| **201** | Nur et al. (2012) | Survey  (Turkey) | Turkish women | 1844  100% female | Conflict Tactic Scales (CTS2) | Physical violence, sexual violence | **Mental distress**  General Health Questionnaire (GHQ-12) |
