# Supplementary material for: Global Prevalence and Mental Health Outcomes of Intimate Partner Violence Among Women: A Systematic Review and Meta-Analysis
Source: Trauma Violence Abuse. 2023 Feb 24;25(1):494–511. doi: 10.1177/15248380231155529 (PMC10666489; doi:10.1177/15248380231155529)
Supplement: sj-docx-3-tva-10.1177_15248380231155529 – Supplemental material for Global Prevalence and Mental Health Outcomes of Intimate Partner Violence Among Women: A Systematic Review and Meta-Analysis [file sj-docx-3-tva-10.1177_15248380231155529.docx]

**Text S1: Example search strategy**

**Database: Ovid MEDLINE(R) without Revisions <1996 to November 2020>**

--------------------------------------------------------------------------------

1 exp Domestic Violence/ (32702)

2 exp Spouse Abuse/ (6065)

3 exp Intimate Partner Violence/ (8264)

4 exp Gender-Based Violence/ (207)

5 Exposure to Violence/ (727)

6 Physical Abuse/ (603)

7 Rape/ (4047)

8 ((abuse* or abusive or assault* or aggress* or batter* or coerci* or controll* or violen* or threat* or manipulati* or maltreat*) adj3 (physical* or sexual* or domestic or emotional* or psychological* or partner* or finan* or econom*)).ti,ab. (41690)

9 (polyvictimi* or poly victim*).ti,ab. (181)

10 (rape* or raping).ti,ab. (7235)

11 or/1-10 (64409)

12 Battered Women/ or "battered wom?n".ti,ab. (2633)

13 Sexual Partners/ (14989)

14 Spouses/ (9036)

15 (spouse or spousal or intima* or relationship* or men or women or wife or wives or husband* or boyfriend* or girlfriend* or couple*).ti,ab. (1825136)

16 or/12-15 (1830704)

17 exp Mental Health/ (30375)

18 exp Mental Disorders/ (813467)

19 exp anxiety disorders/ or exp "bipolar and related disorders"/ or exp mood disorders/ or exp neurotic disorders/ or exp personality disorders/ or exp "schizophrenia spectrum and other psychotic disorders"/ or exp psychotic disorders/ or exp schizophrenia/ or exp sexual dysfunctions, psychological/ or exp "trauma and stressor related disorders"/ or exp stress disorders, traumatic/ (271409)

20 psych*.ti,ab. (486729)

21 depress*.ti,ab. (282127)

22 (anxiet* or anxious).ti,ab. (138444)

23 suicid*.ti,ab. (50414)

24 mood*.ti,ab. (54328)

25 stress*.ti,ab. (575240)

26 schizophreni*.ti,ab. (73587)

27 (mania or manic).ti,ab. (9807)

28 "post traumatic stress disorder*".ti,ab. (8132)

29 PTSD.ti,ab. (18469)

30 "complex trauma*".ti,ab. (397)

31 (bipolar or bi polar).ti,ab. (42042)

32 phobi*.ti,ab. (7007)

33 panic*.ti,ab. (12866)

34 "self harm".ti,ab. (4018)

35 "self injur*".ti,ab. (3224)

36 (social adj3 (function* or withdraw* or isolat*)).ti,ab. (24365)

37 re-experiencing.ti,ab. (530)

38 flashback*.ti,ab. (394)

39 nightmare*.ti,ab. (1843)

40 hyperarousal.ti,ab. (1361)

41 "mood dysregulation".ti,ab. (260)

42 guilt*.ti,ab. (6104)

43 ((sleep* or concentrat*) adj3 (difficult* or problem* or troubl*)).ti,ab. (10165)

44 insomnia*.ti,ab. (14536)

45 "intrusive thought*".ti,ab. (688)

46 intrusion.ti,ab. (4394)

47 hallucinat*.ti,ab. (8774)

48 (delusion* or delud*).ti,ab. (6479)

49 "hearing voice*".ti,ab. (203)

50 "thought disorder*".ti,ab. (824)

51 hopeless*.ti,ab. (3891)

52 self-esteem.ti,ab. (14250)

53 bulimi*.ti,ab. (5405)

54 purging.ti,ab. (2783)

55 "eating disorder*".ti,ab. (14473)

56 anorexi*.ti,ab. (20017)

57 "binge eat*".ti,ab. (4372)

58 ("borderline personality disorder" or BPD).ti,ab. (8988)

59 ("emotionally unstable personality disorder*" or EUPD).ti,ab. (32)

60 ((drug* or alcohol or substance*) adj3 (use* or using or misuse* or misusing or abus*)).ti,ab. (220047)

61 or/17-60 (1925413)

62 11 and 16 and 61 (15453)

63 Case-Control Studies/ or Control Groups/ or Matched-Pair Analysis/ or ((case* adj5 control*) or (case adj3 comparison*) or control group*).ti,ab. (603748)

64 cohort studies/ or longitudinal studies/ or follow-up studies/ or prospective studies/ or retrospective studies/ or cohort.ti,ab. or longitudinal.ti,ab. or prospective.ti,ab. or retrospective.ti,ab. (1995219)

65 Controlled Before-After Studies/ or Controlled Before-After.ti,ab,kw. (693)

66 historically controlled study/ or historically controlled.ti,ab,kw. (301)

67 ("clinical trial" or "clinical trial, phase i" or "clinical trial, phase ii" or clinical trial, phase iii or clinical trial, phase iv or controlled clinical trial or "multicenter study" or "randomized controlled trial").pt. or double-blind method/ or clinical trials as topic/ or clinical trials, phase i as topic/ or clinical trials, phase ii as topic/ or clinical trials, phase iii as topic/ or clinical trials, phase iv as topic/ or controlled clinical trials as topic/ or randomized controlled trials as topic/ or early termination of clinical trials as topic/ or multicenter studies as topic/ or ((randomi?ed adj7 trial*) or (controlled adj3 trial*) or (clinical adj2 trial*) or ((single or doubl* or tripl* or treb*) and (blind* or mask*))).ti,ab,kw. or ("4 arm" or "four arm").ti,ab,kw. (1245465)

68 Cross-Sectional Studies/ or Prevalence/ or (cross-sectional or prevalence or transversal).ti,ab,kw. (815913)

69 Interrupted time series/ or Interrupted time series.ti,ab,kw. (2511)

70 exp Epidemiologic Studies/ (2122380)

71 or/63-70 (3767805)

72 exp Checklist/ or exp "Surveys and Questionnaires"/ or exp "Severity of Illness Index"/ or exp Therapeutic Index/ or exp Outcome Assessment, Health Care/ or exp "Outcome and Process Assessment, Health Care"/ or exp Patient Outcome Assessment/ or exp Symptom Assessment/ or exp Neuropsychological tests/ (2000243)

73 (score* or scoring or scale* or outcome* or report* or tool* or "check list*" or checklist* or inventory or question* or assess* or measure* or test* or index or screen* or evaluat*).ti,ab,kw. (8633448)

74 72 or 73 (9047760)

75 62 and 71 and 74 (7415)

76 limit 75 to (english language and yr="2012-Current") (3777)

77 exp animals/ not humans.sh. (2415897)

78 76 not 77 (3777)

***************************
